# Antimicrobial and cytotoxic activities of natural (Z)-13-docosenamide derived from *Penicillium chrysogenum*

**DOI:** 10.3389/fcimb.2025.1529104

**Published:** 2025-02-27

**Authors:** Nashwa El-Gazzar, Lekaa Said, Fatimah Olyan Al-Otibi, Mohamed Ragab AbdelGawwad, Gamal Rabie

**Affiliations:** ^1^ Department of Botany and Microbiology, Faculty of Science, Zagazig University, Zagazig, Egypt; ^2^ Botany and Microbiology Department, College of Science, King Saud University, Riyadh, Saudi Arabia; ^3^ Genetics and Bioengineering Department, Faculty of Engineering and Natural Sciences, International University of Sarajevo, Sarajevo, Bosnia and Herzegovina

**Keywords:** *Penicillium chrysogenum*, natural antimicrobial products, fatty amide, HPLC, NMR, antimicrobial resistance, antioxidant activity, anticancer activity

## Abstract

**Introduction:**

The synthesis of natural compounds with strong biological activity from affordable sources has proven challenging for scientists. As a natural resource rich in a variety of bioactive substances, fungal metabolites have the potential to be used in medical applications to serve a global purpose towards a sustainable future.

**Methods:**

A total of 25 filamentous fungi were isolated, and their secondary metabolites were assessed for their antimicrobial efficiency.

**Results:**

The extracellular extract of the strain *Penicillium chrysogenum* Pc was selected for its high bioactivity compared with the other whole isolates. The GC-MS analysis of the extracellular extract of *P. chrysogenum* Pc was found to contain approximately 16 variable compounds. After several separation and purification processes using flash chromatography, HPLC, TLC, NMR, and FTIR, the most bioactive compound was identified as (Z)-13-docosenamide or erucylamide with a molecular formula of C22H43NO and a molecular weight of 337.0. The purified (Z)-13-docosenamide possessed antimicrobial activity with an MIC of approximately 10 μg/mL for the tested pathogenic bacteria (*Bacillus subtilis, Staphylococcus aureus, Klebsiella pneumoniae*, and *Escherichia coli*), and 20 μg/mL against the tested fungi (*Penicillium aurantiogriseum* and *Aspergillus fumigatus*). Furthermore, MTT assay showed that (Z)-13-docosenamide inhibited cellviability and the proliferation of hepatocellular carcinoma, in vitro, with an IC {sb}{/sb}50 of 23.8 ± 0.8 μg/mL.

**Conclusion:**

The remarkable bioactivity of (Z)-13- docosenamide makes it a potential candidate to assist the pipeline for the creation of antibacterial and anticancer drugs, which will help to reduce the incidence of antimicrobial resistance (AMR) and fatalities related to cancer.

## 1 Introduction

Antimicrobial resistance (AMR) and cancer constitute a worldwide health threat due to the increase of associated mortality rates (Abdel-Shafi et al., 2020; Enan et al., 2020). Therefore, there is an emergent need to improve, develop, and investigate new antimicrobial and anticancer agents. In this context, natural products are considered safe and more efficient sources of antimicrobial and antitumor substances (Roünsberg et al., 2013; Sitohy et al., 2021; El-Gazzar and Enan, 2020).

Fungal secondary metabolites involve several bioactive substances with antimicrobial, antitumor, anti-inflammatory, immunosuppressive, and antioxidant properties (Jakubczyk and Dussart, 2020; Abo Nahas et al., 2021; Rahman et al., 2022). The vast majority of these compounds (including terpenes glycosides, volatiles, sterols, phenolic compounds, flavonoids, coumarins, lignans, stilbenes, tannins, alkaloids, glucosinolates, amides, and fatty acids) are already employed in the medical field (Simmons et al., 2005; Suryanarayanan et al., 2012; Alamgir et al., 2018; Tetali, 2019; Alam et al., 2021). In this regard, recent studies investigated new natural products that originated from filamentous fungi through numerous fermentation techniques (El-Sayed et al., 2020; Robey et al., 2021).

The fungal extracellular or intracellular extracts are a natural resource for the discovery of new derivatives with useful bioactivity that could have further medicinal use (Suryanarayanan et al., 2009; Devi et al., 2020; Robey et al., 2021). Al-Romaizan (2019) reported that filamentous fungi generate an extensive variety of medicinal secondary metabolites, including benzopyrones derivatives, phenolic acids, alkaloids, xanthones, flavonoids, tetralones, terpenoids, quinones, steroids, and others. These bioactive metabolites have a variety of applications, including antibiotic development, antiparasitic, agrochemicals, and anticancer medications. Numerous biochemical and pharmacological investigations have demonstrated that fatty acid amides, which belong to the endo-cannabinoid family, constitute a novel class of physiologically significant lipids. Research using synthetic fatty acid amides demonstrated antiproliferative action against a number of tumor cells, indicating that variations in antiproliferative profiles could be caused by variations in the fatty acid moieties on groups linked to the nitrogen atom. The binding to cannabinoid and vanilloid receptors revealed the impact of fatty acid amides and endo-cannabinoids on the growth of cancer cells. Additionally, the fatty acid chain plays a crucial role in biological activity, probably because it makes bacterial cells more permeable. One of the critical findings is the discovery of new derivatives, such as (Z)-13-docosenamide, with useful bioactivity that could have further medicinal use.

(Z)-13-docosenamide known as erucamide is a fatty amide derivative as well as a bovine mesentery angiogenic lipid (Wakamatsu et al., 1990). Its receptor-mediated control of physiological activities in higher animals remains unclear, but seems to be significant (Hamberger and Stenhagen, 2003). Glycerides of euric acid, the monounsaturated fatty acid precursor of eurcamide (13-docosenamide), are found in large quantities in mustard and rapeseed seeds (Awasthi and Singh 2007). Moreover, some studies showed that the presence of fatty amide such as (Z)-13-docosenamide provides significant antimicrobial, antioxidant, and anticancer properties (Gueule et al., 2015; Naim et al., 2016; Ker et al., 2017; Xie et al., 2021). (Z)-13-docosenamide involves notable anticancer activity against adenocarcinomic human alveolar basal epithelial cells (A549) (Priya et al., 2018). The antibacterial, anti-inflammatory, and antioxidant properties of several common phytoconstituents, such as (Z)-13-docosenamide, quercetin, phytol, 1-hexadecanol, and 1-hexadecene, are necessary for the acute and chronic wound healing processes (Prasathkumar et al., 2022). Based on the findings of previous studies and the difficulties that faced scientists in synthesizing natural substances with potent biological activity from cost-effective sources, the main objectives of this study are to isolate filamentous fungi from the soil and to separate the active compounds immediately in the secondary products of the most potent fungus. The present work primarily investigated the purification and evaluation of the antimicrobial and antiproliferative activities of the new natural fungal compound.

## 2 Materials and methods

### 2.1 Sample collection and isolation of filamentous fungi

Approximately 15 rhizosphere samples were obtained from diverse areas in Sharkia Governorate, Egypt. Soil samples were transported to the laboratory on the same day. The spread plat method was used for the isolation of filamentous fungi using Czapek Dox Agar (CDA) (Hi Media Lab. Pvt. Ltd. Ref. GM075) and potato dextrose agar (PDA) plates. Soil samples were dispersed on the plates’ surface, and the plates were then incubated at 28–30°C for 5–7 days. In order to obtain pure fungal isolates, the colonies that emerged were then sub-cultivated on sterile PDA plates.

### 2.2 Morphological and molecular identification of filamentous fungi

The fungal isolates were subsequently identified using their morphological characteristics according to Thom and Raper (1951), Kenneth et al. (1965), and Sutton (1980). Next, the isolate that produced the most bioactive secondary metabolites was confirmed by molecular identification of the 18–28S rRNA gene at SolGent Company in Daejeon, South Korea. PCR amplification of the 18–28S rRNA gene was performed using the forward and reverse primers ITS1 (5′-TCCGTAGGTGAACCTGCGG-3′) and ITS4 (5′-TCCTCCGCTTATTGATATGC-3′). The same primers were used for sequencing the purified PCR amplicons (White etal., 1990). The similarities of the obtained sequences to the nucleotide sequences in GenBank were searched using the BLAST (BLASTn) search tool. The sequences were then deposited in GenBank after obtaining an accession number. MegAlign (DNA Star) software version 5.05 was used for the phylogenetic analysis of the sequences.

### 2.3 Extraction of secondary metabolites

The obtained pure fungal isolates were inoculated separately onto sterile PDA slants and then incubated until sporulation at 28°C for a week. Then, sterile distilled water (10 mL) was added, and spore suspension was made by slightly expelling spores from conidiophores using a sterile inoculation loop. To remove the mycelial debris, four layers of sterile cheesecloth were used to filter the spore solution followed by filtration with a Whatman No. 1 filter paper. Sterile 0.1% (v/v) peptone water was used to prepare serial spore dilutions. The inoculum level was adjusted at 4×10^2^ cells/mL using a hemocytometer for spore counting (Ellis et al., 1991). Secondary metabolites were extracted as follows: approximately 1 mL of 4×10^2^spores/mL of each fungal isolate was added separately into 100 mL of sterile PDA. After static incubation at 28°C for 21 days (El-Gazzar and Ismail, 2020), the cultures were filtered. The intracellular secondary metabolites were extracted by grinding the fungal mycelial mat with sterile fine sand and chloroform–methanol (2:1, v/v). Then, the homogenate was centrifuged, filtered, and air-dried until the solvent has completely evaporated. Methanol was then used to dissolve the metabolites and the mixture was kept at 5°C. Afterwards, using a separating funnel, the broth filtrate was treated with chloroform–methanol (2:1, v/v), shaken properly, and left for 6 h at room temperature until complete separation. The residue was fully extracted twice using methanol, which was also used as a solvent for dissolving the metabolites, and the mixture was maintained at 5°C (Abdel-Salam et al., 2001).

### 2.4 Biological activity of crude secondary metabolites

#### 2.4.1 Antioxidant activity of crude secondary metabolites

1,1-Diphenyl-2-picryl hydrazyl (DPPH) was used as a benchmark for detecting the antioxidant effectiveness of the crude compounds of the isolated filamentous fungi (Baba and Malik, 2015). Concisely, DPPH solution (3.8 mL) was mixed with 10 μL of each extract (50 μg/mL) and incubated at 37°C for 30 min. The absorbance was then measured at 517 nm, using ascorbic acid as a positive control; all experiments were conducted in triplicate. The scavenging efficacy rate was then calculated (Baba and Malik, 2015). The antioxidant efficiency of the purified active compound was determined with the same procedure.

#### 2.4.2 Antimicrobial activity of crude secondary metabolites

The antimicrobial efficiency of the crude intracellular and extracellular secondary products was assessed against four bacterial species, namely, *Staphylococcus aureus* ATCC 25923, *Bacillus subtilis* RCMB 015s (1) NRRL-543, *Escherichia coli* ATCC25922, and *Klebsiella pneumoniae* RCMB003 (1) ATCC13883, and two fungal species, namely, *Aspergillus fumigatus* RCMB002008 and *Penicillium aurantiogriseum* IMI89372. The test strains were received from Al-Azhar University’s Regional Centre for Mycology and Biotechnology in Cairo, Egypt. The agar well diffusion technique was used to determine the crude secondary metabolites’ antimicrobial activity of the isolated filamentous fungi (Enan et al., 2013, 2020). Plates of PDA for fungi and nutrient agar for bacteria were inoculated with ∼10^5^ CFU/mL of the bacterial test strains and 2.0×10^6^ spores/mL of the fungal test strains. Afterwards, 6-mm agar wells were then loaded with approximately 100 µL of the crude secondary metabolite extract (200 μg/mL). Methanol (the solvent) served as a negative control, ketoconazole was employed as an antifungal positive control, and gentamycin was used as an antibacterial positive control. To allow the crude extracts to diffuse through the agar, the plates were maintained for an hour in a refrigerator. Next, the plates inoculated with bacteria were incubated for 24 h at 37°C, while the plates seeded with fungi were incubated at 28°C for 5–7 days.

#### 2.4.3 TLC analysis of crude secondary metabolites

Thin-layer chromatography (TLC) was used to separate the secondary metabolites of the tested fungi (Dedio et al., 1969). The crude extract was spotted on TLC sheets (silica gel G-60 aluminum sheet, Merck, Germany) using the mobile phase system chloroform:methanol (9:1 v/v) (El-Sayed et al., 2020). The sheets were evaluated using the CAMMAG LINOMAT-5 application system to optimize the most advanced solvent method for separating the crude extract into distinct metabolites.

### 2.5 Separation and purification of bioactive compounds

The crude extract that possessed the highest antioxidant and antimicrobial activity was then analyzed using several techniques to separate, purify, and identify the bioactive compounds.

#### 2.5.1 GC-MS analysis

GC-MS (Thermo-scientific trace 1310 Gas chromatograph connected to an ISQ LT single quadrupole mass spectrometer at the Regional Centre for Mycology and Biotechnology) was used to analyze the chemical composition of the bioactive compounds. The column was a fused capillary column (DB1 J&W; 30 m length, 0.25 mm inner diameter, and 1.5 μm film thickness) coated with dimethyl polysiloxane. The separated peaks were detected using the WILEY mass spectral database (Enan et al., 2020; Osman et al., 2021).

#### 2.5.2 Flash chromatography

To obtain better separation and purification of the secondary products, a flash chromatography system (PuriFlash 4100 system; Interchim; Montluçon, France) was employed. The TLC was performed as aforementioned to separate the secondary metabolites of the selected fungus (Al-Azzawi et al., 2022). The resulting bands from the TLC experiment were prepared, with each band being a separate specimen. Specimens were dispersed in 50 mL of methanol, and then they were loaded onto a column with 12 g of silica column (25 g - flash - NP column 30 μm). After several runs, the final concentrated fraction was obtained. Then, it was dissolved in methanol and inoculated into the Reverse Silica C18 column. Hexane and ethyl acetate were the solvents used in the system (A). The flow rate was adjusted at 10 mL/min and the ramp mode started with the solvent (A) alone in the first minute and then elevated to 60%. Next, the solvent (B) was used and run for 7 min, and then it was raised to 65%, with a 1-min hold period following every 1% increase of the solvents (Al-Azzawi et al., 2022). The flash chromatography system embraced a sample loading module, a fraction collector, a PDA-UV-Vis detector 190–840 nm, and a combining HPLC quaternary pump for preparative separations. To monitor the process and manage the system, the Interchem Software 5.0 was applied. Furthermore, the purified compounds from flash chromatography were also applied on TLC and HPLC to detect their purity.

#### 2.5.3 HPLC analysis

HPLC analysis was used to identify the compounds found in the previous stage. The sample was diluted in 1 mL of methanol and sonicated for 15 min; then, it was filtered using a 0.22-μm Millipore filter. Subsequently, approximately 100 μL of the solution was injected into an HPLC system (Waters 2690 Alliance HPLC system with a Waters 996 photodiode array detector). The HPLC analysis was carried out using a C18 column (Kromasil 1 cm × 25 cm, 5 μm). For elution of methanol and water gradient, the mode of the mobile phase was adjusted at a flow rate of 3 mL/min at a wavelength of 254 nm at room temperature (El-Sayed et al., 2020).

#### 2.5.4 NMR and FTIR spectral analysis

Nuclear magnetic resonance (NMR) spectral analysis and Fourier-transform infrared (FTIR) spectral analysis were conducted to elucidate the chemical structure of the purified compound (Abdel-Shafi et al., 2020; Osman et al., 2021; Singab et al., 2021). The ^1^H-NMR and C-NMR spectral analysis was performed using Bruker Ascend 400/R (Bruker^®^, AVANCE III HD, 400 MHz, Zürich, Switzerland) at the Drug Discovery Center, Research and Development, Faculty of Pharmacy, Ain Shams University. The sample was dissolved in chloroform. Hertz (Hz) and parts per million (δ-scale) were used to describe the coupling constants and chemical shifts, correspondingly. The FTIR analysis was performed using Bruker Spectrometer FT-IR in the 400–4,000 cm^−1^ spectrum with KBr pellets formed into discs under vacuum (at the Micro-Analytical Centre, Cairo University, Egypt).

### 2.6 Antioxidant activity of the most potent (Z)-13-docosenamide

DPPH free radicals were used to measure the antioxidant effectiveness of (Z)-13-docosenamide using a positive reference of ascorbic acid as mentioned above (Baba and Malik, 2015).

### 2.7 Antimicrobial activity of (Z)-13-docosenamide

The purified bioactive compound [(Z)-13-docosenamide] was prepared in methanol as a stock solution (200 µg/mL), maintained at 5°C. The agar well diffusion method was employed, as noted, for testing the antimicrobial efficiency of the purified bioactive secondary metabolite and determining its MIC (minimal inhibition). The bactericidal efficiency was tested against 10 bacterial species [5 of which were Gram-positive, namely, *Staphylococcus aureus* ATCC 25923, *Bacillus subtilis* RCMB 015s (1) NRRL-543, *Staphylococcus pyogenes* ATCC 19615, *Listeria monocytogenes* LMG 10470, and *Listeria innocua* LMG11387, and 5 were Gram-negative, namely, *Salmonella typhimurium* RCHB006 (1) ATCC14028, *Pseudomonas aeruginosa* LMG 8029, *Enterobacter cloacae* RCMB 001 ATCC23355, *Escherichia coli* ATCC25922, and *Klebsiella pneumoniae* RCMB003 (1) ATCC13883] and three fungal species (*Aspergillus fumigatus* RCMB002008, *Penicillium aurantiogriseum* IMI89372, and *Candida albicans* RCMB 005003 ATCC 10231).

#### 2.7.1 MIC determination

To determine the MIC, different dilutions (5, 10, 20, 30, 40, 50, 70, 80, 90, and 100 g/mL) of the bioactive secondary metabolite of *Penicillium chrysogenum* were tested separately against the test strains. Each concentration was repeated thrice for each test strain. The plates were incubated for 1 day at 37°C for bacteria and for 1 week at 28°C for fungi. Then, the plates were observed for the presence or absence of inhibition, the diameters of the resulting inhibition zones were measured and recorded, and the MIC was determined (Abdel-Shafi et al., 2020; Abou Elez et al., 2023).

#### 2.7.2 TEM examination

Transmission electron microscopy (TEM) was used to detect the effects of (Z)-13-docosenamide on the ultrastructure of *K. pneumoniae* and *P. aurantiogriseum*. Fresh broth cultures of *K. pneumoniae* (10^6^ CFU/mL) and *P. aurantiogriseum* (2.0×10^6^ spores/mL) were treated separately with the corresponding MICs and were then incubated for 4 h at 37°C and 28°C, respectively. Afterwards, the cultures were centrifuged for 10 min at 4,000 rpm, and then the cultures were centrifuged to obtain the cells. The pellets were washed twice with distilled water and fixed with 3% glutaraldehyde and potassium permanganate for 5 min at room temperature. Next, the pellets were washed with phosphate buffer. Then, the specimens were dehydrated with absolute ethanol for 30 min. Finally, the samples were immersed in pure resin and sliced into thin slices. The slices were loaded onto TEM copper grids and stained twice with lead citrate and uranyl acetate to be examined using TEM (JEOL JEM-1010, Tokyo, Japan) (Abou Elez et al., 2023).

### 2.8 Anticancer activity of (Z)-13-docosenamide

#### 2.8.1 MTT assay

The MTT colorimetric assay was performed at VACSERA Tissue Culture Unit, as described by Gomha et al. (2015) and Mosmann (1983), to test the anticancer activity of the bioactive secondary metabolite against human hepatocellular carcinoma (HepG-2) cells, the human breast cancer cell line (MCF-7 cells), and the human lung carcinoma cell line (A549 cells). In addition, the mouse liver cell line (NBL cells) and the human lung fibroblast normal cell line (WI-38) were obtained from the American Type Culture Collection (ATCC, Rockville, MD). The tumor cells were trypsinized and washed with sterile phosphate-buffered saline (PBS). Next, the cells were inoculated into a 96-well plate at a final cell density of 10^4^ cells/100 µL, using RPMI-1640 enriched with 10% fetal bovine serum (FBS). Then, the plate was incubated at 37°C in the presence of 5% CO_2_ for 24 h. Afterwards, the medium was replaced with FBS-free media supplemented with different dilutions of the bioactive compound (3.90, 7.80, 15.60, 31.25, 62.50, 125, 250, and 500 µg/mL). After 48 h of incubation at 37°C and 5% CO_2_, the wells were cleaned with PBS. After adding 50 µL of MTT reagent [3-(4,5-dimethylthiazol-2-yl)-2,5-diphenyltetrazolium bromide; 0.5 mg/mL] to each well, the plate was allowed to rest for 4 h. After discarding the supernatant from each well, the precipitate of dark blue formazan crystals was dissolved in 50 µL of dimethyl sulfoxide (99.9%) followed by stirring. The supernatants were then discarded, and the live cell count was determined by measuring the absorbance of formazan at 490 nm with a microplate reader (Sunrise, TECAN, Inc., USA). To calculate the rate needed to exhibit a harmful effect on 50% of intact cells, GraphPad Prism software (San Diego, CA, USA) was applied (Pandey et al., 2023). The rate of viability was determined as follows:


$$\%\text{ Cell viability }=\;\frac{\text{Mean O.D of treated cells}}{\text{Mean O.D of untreated cells}}\;\times \;100$$

#### 2.8.2 Cytotoxicity evaluation and determination of oxidative enzymes

##### 2.8.2.1 Antioxidant assay using ABTS scavenging capacity

The ABTS scavenging capacity technique is a decolorization assay that assesses antioxidants’ ability to react directly with ABTS radicals produced by a chemical process (Re et al., 1999). The antioxidant activity was measured using the ABTS radical scavenging technique, as reported by others (Sánchez et al., 2007; Ling et al., 2009). ABTS radical cation (ABTS+) was created by mixing a 1.8 mM ABTS stock solution (Sigma, PN: A3219) with 0.63 mM potassium persulfate and leaving the mixture to rest in the dark at room temperature for 12–16 h before use. The solution was diluted with ethanol until absorbance reached 0.700 (± 0.030) at 734 nm. At room temperature, samples or extracts were diluted 1:10 with methanol (80%). Then, 190 μL of radical solution was mixed with 10 μL of diluted extracts in a microtiter plate. The absorbance at 734 nm was measured every 1 min until 13 min after initial mixing. Appropriate solvent blanks were run in each assay. Ascorbic acid and methanol were used as the standard antioxidant and negative control, respectively. Experiments were performed three times with three replicates for each sample. The percent free radical scavenging activity was calculated according to the following formula:


$$\%\text{ Free radical scavenging activity }=\;[(A_{n}\;-\;A_{s})]\;\times \;100]/A_{n}$$

where *A*_n_ is the final absorbance values of the negative control and *A*_s_ is the final absorbance values of the sample.

##### 2.8.2.2 Propagation of carcinoma cell lines

The cell lines were grown in RPMI-1640 media. The cancer cell lines were cultured in a 75-cm^3^ culture Corning^®^ flask at 37°C for 48 h in a humidified water jacketed CO_2_ incubator (Shel Lab, Sheldon Manufacturing, Inc., USA). An inverted microscope (CKX41; Olympus, Japan) was used to examine the cell monolayer and ensure the lack of bacterial and fungal contamination. To count the number of cells, the cell monolayer was rinsed with 5 mL of PBS free of Ca2+/Mg2+, then 2.5 mL of 0.53 mM trypsin/EDTA solution was added to the culture flask and incubated for 7–15 min. After the cells were removed from the flask, 6 mL of maintenance medium was added to inhibit the activity of trypsin. A hemocytometer was used to determine the number of viable cells using trypan blue staining (Wilson, 2000).

##### 2.8.2.3 Quantification of oxidative stress

To investigate the effect of oxidative stress on tumor cell lines, the most impacted cell line (HepG-2 cells) was chosen for further testing. HepG-2 cells were planted at 5×10^5^ cells/mL in 24-well tissue culture plates. After forming a monolayer cell sheet in each well, the tested substance was distributed into a 24-well tissue culture plate at 23.8 µg/mL, based on 50% inhibitory concentration (IC_50_) values, including wells for cell controls. Each treatment was done in triplicate. Following treatment, the growth media was evacuated and the cells were extracted from each well.

##### 2.8.2.4 Preparation of the cell lysate

After incubation, trypsin was used to extract the cells. A 2-mL culture medium was added and the combination was centrifuged at 1,500 rpm for 10 min at 4˚C in a Sigma refrigerated centrifuge. The mixture was then rinsed with PBS (pH 7.4) three times. The pellet was immersed in an extraction solution that comprised 20 mM of potassium phosphate buffer (pH 7) and a protease inhibitor cocktail. The cells were sonicated in ice-cold normal saline (1/9, w/v) in a Virsonic^®^ ultrasonic cell disruptor for 10 min, then centrifuged at 5,000 rpm for 5 min at 4˚C, and the supernatant was kept at −80˚C until the experiments were done (Timur et al., 2005; Alarifi, 2011).

##### 2.8.2.5 Protein estimation

According to Bradford’s (1976) method, protein measurement was performed on each sample using bovine serum albumin as a reference standard. After adding 100 µL of protein sample or standard (in duplicate) to each well of the 96-well microplate, the Bradford reagent was added with thorough mixing and allowed to sit for 5 min. Following the incubation times, the absorbance at 595 nm was measured. Protein concentration was determined by performing a regression of series standards (bovine serum albumin, concentration 0–100 µg/mL) on the same 96-well microplate.

##### 2.8.2.6 Determination of Glutathione content

With minor adjustments, the method of Siddiqui et al. (2010) was used to determine the intracellular glutathione (GSH) content. To summarize, 1 mL of sonicated cell suspension was mixed with 1 mL of 10% trichloroacetic acid (TCA) and allowed to sit on ice for 1 hour in order to fully precipitate the proteins. The mixture was then centrifuged for 10 min at 3,000 rpm. After adding 0.02 M ethylenediaminetetraacetic acid (EDTA) and 0.01 M 5,5′-dithionitrobenzoic acid (DTNB) to 2 mL of 0.4 M Tris buffer (pH 8.9), the supernatant was diluted with 0.5 mL of distilled water to reach a final volume of 3 mL. The tubes were shaken in a water bath at 37°C for 10 min. At 412 nm, the absorbance of yellow color was developed using a microplate reader (SunRise, TECAN, Inc., USA).

##### 2.8.2.7 Activity of catalase

Following Sinha’s (1972) approach, catalase activity was measured using hydrogen peroxide (H_2_O_2_) as a substrate. The reaction mixture, which had a final volume of 1 mL, contained enzyme protein, 0.08 µmol of H_2_O_2_, and phosphate buffer (pH 7.0). The enzyme activity was determined using a UV-Visible Spectrophotometer (Spectronic, Milton Roy, UK) when H_2_O_2_ vanished at 570 nm.

##### 2.8.2.8 Superoxide dismutase activity

The method outlined by Kakkar et al. (1984) was used to measure the superoxide dismutase (SOD) activity. Nicotinamide adenine dinucleotide (NADH) was added to a final volume of 3 mL that contained 0.052 M sodium pyrophosphate buffer (pH 8.3), 186 µM phenozine methosulfate (PMS), 300 µM nitroblue tetrazolium (NBT), 780 µM NADH, sonicated enzyme preparation, and water. The reaction was then initiated and incubated for 90 s at 37°C. Following the addition of 1.0 mL of glacial acetic acid to halt the reaction, 4.0 mL of n-butanol was used to vigorously shake the contents. After letting the mixture stand for 10 min, the butanol layer was separated by centrifugation. At 560 nm, the chromogen’s color intensity in butanol was measured against butanol using a spectrophotometer. A mixture devoid of enzyme containing cell suspension served as control.

##### 2.8.2.9 Malondialdehyde assay

The last indicator of the lipid peroxidation pathway is malondialdehyde (MDA), which is tested using this method. According to Buege and Aust (1978), this assay is based on the reaction of MDA with thiobarbituric acid (TBA), which results in the MDATBA adduct that may be measured calorimetrically. To put it briefly, cells were gathered by centrifugation, sonicated in 1.15% ice-cold potassium chloride, then centrifuged for 10 min at 3,000 rounds per minute. In a boiling bath, the resultant supernatant (1 mL) was heated to 100°C for 15 min after being combined with 2 mL of TBA reagent (15% TCA, 0.7% TBA, and 0.25 N HCl). After being chilled, the material was centrifuged for 10 min at 1,500 rpm; 535 nm was used to test the supernatant’s absorbance.

#### 2.8.3 Evaluation of the mechanism of cytotoxicity against the HEPG2 cell line with ROS determination

The ATCC (Rockville, MD) provided the human liver cancer cell line, HEPG-2. The cells were grown in Dulbecco’s modified Eagle’s medium (DMEM), which was enhanced with 50 µg/mL gentamycin, 10% heat-inactivated fetal bovine serum, 1% L-glutamine, and HEPES buffer. The cells were kept in a humidified environment with 5% CO_2_ at 37°C.

A day prior to the experiment, the HEPG-2 cells were seeded into six-well plates at a density of 5×10^5^ cells/mL in order to investigate the potential mode of action on the cell line.

The active compounds were poured into a six-well tissue culture plate at IC_50_ concentration after a full monolayer cell sheet had formed in each well of the plate. Each therapy was carried out three times. Following incubation, cells were collected using trypsin (0.25%); 2 mL of culture media was then added, and the combination was centrifuged in a Sigma refrigerated centrifuge at 1,500 rpm for 10 min at 4˚C. Three PBS (pH 7.4) washes were then performed. The pellet was put into an extraction solution that included a protease inhibitor cocktail and 20 mM of a potassium phosphate buffer (pH 7). The cells were sonicated in ice-cold normal saline (1/9, w/v) in a Virsonic^®^ ultrasonic cell disruptor for 10 min, then centrifuged at a speed of 5,000 rpm for 5 min at 4˚C and the supernatant was stored at −80˚C until the experiments were done. Additionally, reactive oxygen species (ROS) levels in HEPG-2 cells (treated or untreated) were measured using the previously described ELISA colorimetric kits, according to the manufacturer’s instructions (Eldehna et al., 2018).

### 2.9 *In silico* study of (Z)-13-docosenamide

The study investigated molecular interactions between (Z)-13-docosenamide and three target proteins of HEPG-2, *Klebsiella pneumoniae*, and *Penicillium aurantiogriseum* through *in silico* approaches. Firstly, the ligand structure of (Z)-13-docosenamide (erucylamide, C_22_H_43_NO, MW 337, CAS#112-48-5, Entry#30130) was generated and optimized using ChemBio Office software. The structural integrity and parameters were verified through the DrugBank database to ensure accuracy. The three-dimensional conformation was energy-minimized to achieve a stable state suitable for molecular docking studies. In addition, the target protein structures for this study were obtained from established structural databases. The crystal structure of HEPG-2, transforming growth factor-beta type II receptor (TGF-β II) was retrieved from the Protein Data Bank (PDB ID: 1plo), along with the *Klebsiella pneumoniae*, outer membrane protein A (OMP-A) structure (PDB ID: 2k0l). The *Penicillium aurantiogriseum*, tubulin beta chain protein structure was accessed through UniProt (ID: AF-G5CIU8-F1-model_v4). These structures underwent rigorous preparation protocols to ensure optimal conditions for molecular interactions and subsequent analyses for (Z)-13-docosenamide.

#### 2.9.1 Molecular docking analyses of (Z)-13-docosenamide

Molecular docking analyses were conducted using the AutoDock Vina software to evaluate the binding interactions between the prepared proteins and ligand. Prior to docking simulations, the protein structures underwent essential preparatory steps in UCSF Chimera, including the removal of water molecules and co-crystallized ligands. Polar hydrogen atoms were added to the structures, and partial charges were assigned to create optimal conditions for the docking simulations. The protocol generated multiple binding poses, which were systematically analyzed to calculate binding energies and identify key protein–ligand interactions.

#### 2.9.2 Molecular dynamics simulations of (Z)-13-docosenamide and the target proteins

Following the initial docking analysis, comprehensive molecular simulation studies were performed to evaluate the stability and dynamic behavior of the protein–ligand complexes. The simulation protocol employed energy minimization techniques using UCSF Chimera, with specific parameters designed to optimize the molecular systems. The minimization procedure utilized a combination of steepest descent optimization (500 steps, 0.01-Å step size) and conjugate gradient optimization (50 steps, 0.01-Å step size), maintaining a constant temperature of 298.15 K throughout the simulation process. This rigorous minimization protocol served to eliminate steric clashes within the complexes, optimize the geometry of the protein–ligand interfaces, and achieve stable conformational states for subsequent analyses. The molecular interactions between (Z)-13-docosenamide and the target proteins were analyzed in detail, focusing on multiple aspects of the binding characteristics. The analysis encompassed free binding energy calculations at 298.15 K, the evaluation of potential hydrogen bonding patterns, the assessment of van der Waals interactions, and the characterization of hydrophobic contacts. Particular attention was paid to identifying specific amino acid residues involved in ligand binding.

### 2.10 Statistical analysis

Statistical analysis was performed with SPSS statistical software, version 25. The Shapiro–Wilk test was used to determine whether the data were distributed normally. Results were expressed as the mean ± standard deviation (SD). One-way ANOVA followed by pairwise comparisons determined the difference between the fungal isolate using Tukey test, while independent-samples *t*-tests were used to investigate the changes in variables between the study and control strains. Repeated-measures ANOVA was used to determine the differences between different concentrations with *post-hoc* pairwise comparisons adjusted by Bonferroni adjustment test. *p*-values less than or equal to 0.05 were considered statistically significant (Shapiro and Wilk, 1965; Tukey et al., 1985; Sasaki, 1996; El-Gazzar et al., 2023).

## 3 Results

### 3.1 Identification of fungal isolates

**Figure 1:**
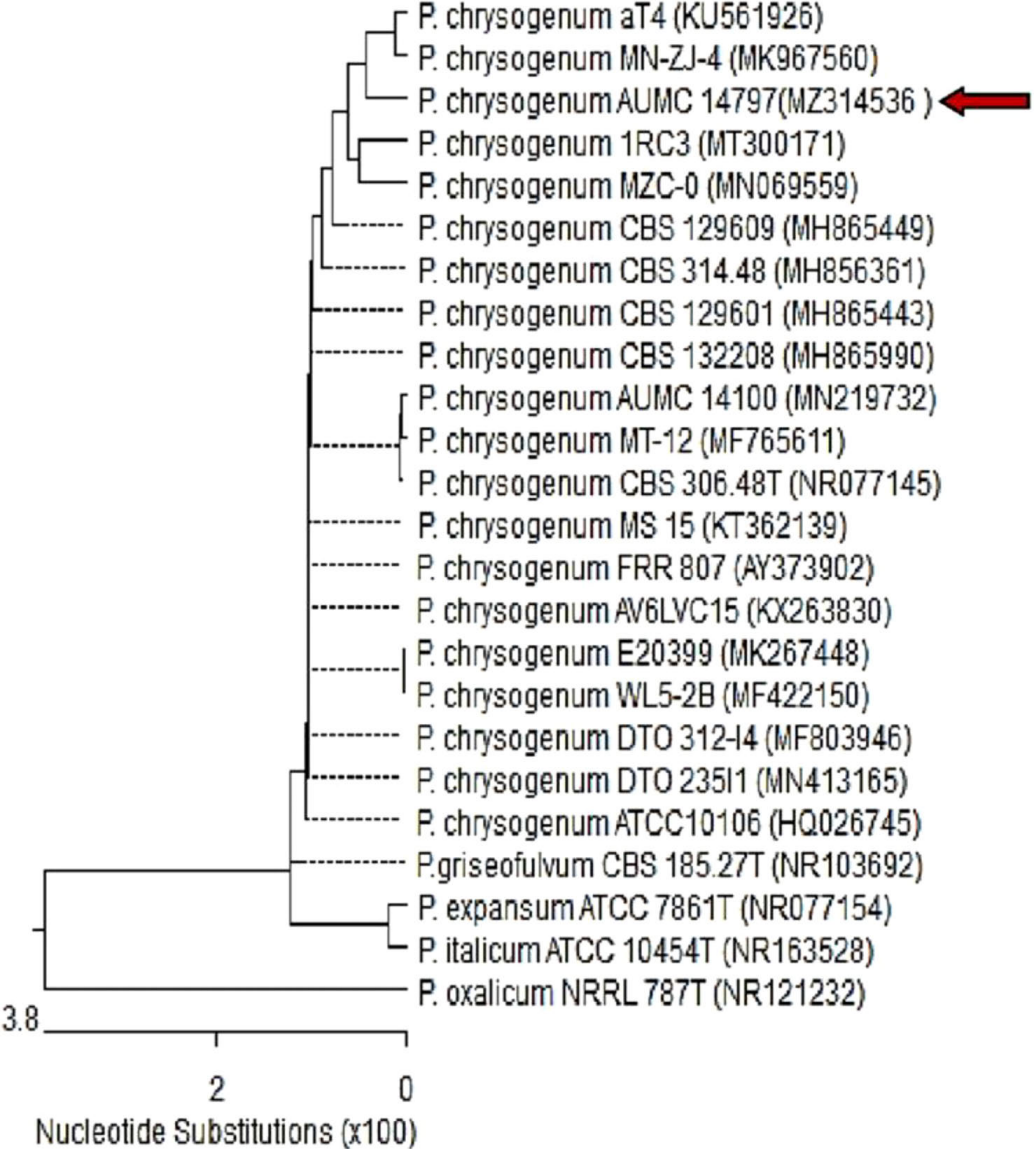
Phylogenetic tree based on ITS sequences of 18S rDNA of the fungal strain isolated in the present study (AUMC14797-MZ314536, arrowed) aligned with closely related sequences accessed from the GenBank (*P*. = *Penicillium*). Strain AUMC14797 showed 100% and 100% identity with several strains of *P. chrysogenum* including the type strain CBS 306.48T (NR_077145). *P. oxalicum* is included in the tree as an outgroup strain.

A total of 25 filamentous fungi were isolated from the collected soil samples. The isolates were initially identified, using an international reference key, according to their morphological characteristics (**Supplementary Table S1**). Approximately 36% of the isolated fungi belong to the *Aspergillus* sp., sp while only three isolates (12%) were found to be of the *Penicillium* sp. The rest of the isolates were primarily identified as *Alternaria* sp., *Rhizopus* sp., sp *Trichoderma* sp., sp *Fusarium* sp., sp *Cunninghamella* sp., sp *Helmithosporium* sp., sp *Ucladium* sp., sp *Mucor* sp., sp *Beauvaria* sp., and *Cladosporium* sp. The whole numbers of isolates were further screened for the potential of their intracellular and extracellular secondary metabolites to possess antioxidant and antimicrobial activity. Subsequently, the fungus that produced the most potent secondary metabolites was genetically confirmed by 18S–28S rRNA gene sequencing. Afterwards, the obtained DNA sequences were submitted to GenBank and searched for similarity with similar sequences in the database. The highest sequence similarity was with the strain *P. chrysogenum* CBS 306.48T (NR_077145). The sequences in the present study were deposited into GenBank (https://www.ncbi.nlm.nih.gov/Genbank) under accession no. MZ314536, and a phylogenetic tree was constructed (**Figure 1**).

### 3.2 Biological activity of the crude secondary metabolites

#### 3.2.1 Antioxidant activity of the crude secondary metabolites

The antioxidant activity of the crude extracellular and intracellular secondary metabolites of the fungal isolates was detected using the DPPH method. As **Supplementary Table S1** shows, both extra- and intracellular secondary metabolites of only 10 isolates (Pc, Af, At, Ao, Cs, Fo, An, Ps, Pi, and Ft) possessed antioxidant activity. The extracellular secondary metabolites of these 10 isolates showed better antioxidant properties than the intracellular ones. Compared to ascorbic acid, the highest antioxidant efficiency was exhibited in the case of the extracellular and intracellular extracts of the fungus *P. chrysogenum* Pc, which was 93.27% for the extracellular extract and 75.87% for the intracellular extract.

#### 3.2.2 Antimicrobial activity of the crude secondary metabolites

The antimicrobial activity of the intracellular and extracellular extracts of all fungal isolates was evaluated using the agar well diffusion technique against *S. aureus, B. subtilis, E. coli, K. pneumoniae, A. fumigatus*, and *P. aurantiogriseum*. As shown in **Supplementary Table S2**, some fungal extracts possessed antimicrobial activity and some had no inhibitory action. The common pattern of the results is the extracellular extracts possessing higher antimicrobial activity than the intracellular extracts. The extracellular metabolites of the isolates *P. chrysogenum* Pc, *A. fumigatus* Au*, A. flavus* Af*, A. terreus* At*, A. oryzae* Ao*, Alternaria* sp. As*, Helminthosporium* sp. Hs, and *Cladosporirum* sp. Cs showed the highest antibacterial and antifungal efficiency against the test strains. The extracellular metabolites of *P. chrysogenum* Pc was the most potent among the tested extracts. The diameter of inhibition zone caused by the intracellular extract of *P. chrysogenum* Pc against *K. pneumoniae* and *S. aureus* was 12.17 and 10.13 mm, respectively, while in the case of the extracellular extract, the diameter of inhibition zone reached 39.23 and 39.7 mm, correspondingly. On the other side, the intracellular extract of *P. chrysogenum* Pc was able to inhibit only *A. fumigatus*, and it had no fungicidal activity against *P. aurantiogriseum*. In contrast, the diameter of inhibition zone in the case of the extracellular extract was even greater than that caused by the positive control (ketoconazole) 33.37 and 36.50 mm against *A. fumigatus* and *P. aurantiogriseum*, compared to 18.43 and 19.23 mm, respectively.

#### 3.2.3 TLC analysis of the crude secondary metabolites

The TLC of the extracellular secondary metabolites of the most active fungi different showed the presence of several bands (**Supplementary Figure S1A**).

### 3.3 Purification and identification of bioactive compounds

#### 3.3.1 GC-MS analysis of the crude extract of *P. chrysogenum*

GC-MS analysis of the extracellular secondary metabolites of the most potent fungus (*P. chrysogenum* Pc) revealed the presence of 16 compounds that are known for their antioxidant, antibacterial, and anticancer activity (**Table 1**, **Supplementary Figure S2**). The detected bioactive secondary metabolites included esters, amides, fatty acids, ketones, aldehydes, phthalates, anhydrides, cholesterols, and pyrazole. Most of these compounds are known for their antibacterial and anticancer potential. Additionally, the presence of unsaturated fatty esters, heterocyclic compounds, pyrazine, and pyridines, which have antimicrobial and antiproliferative properties, was detected.

**Table 1:** The chemical compounds that were detected in the GC-MS analysis of the extracellular extract of *P. chrysogenum* Pc.

Molecular formula	Molecular weight	Compound name	Area	Parent ion (M+)	Base peak (m/e) (100%)	Chemical structure
**C_9_H_7_N**	129.0	Quinoline (Heterocyclic)	0.82	129.0	102.0	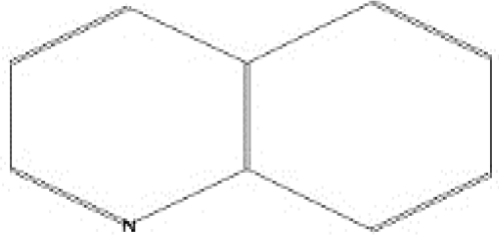
**C_12_H_16_O_5_**	240.0	2-Carboxymethyl-3-n-hexylmaleic acid anlydride (Anlydride)	8.65	240.0	126.0	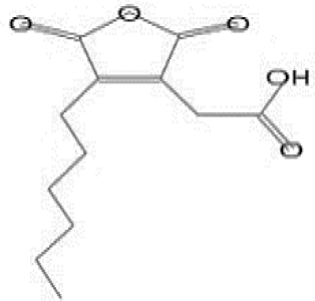
**C_13_H_22_N_2_O**	222.0	1-Methyl-8-propyl-3,6-diazahomoadamantan-9-one (Heterocyclic)	52.48	222.0	58.0	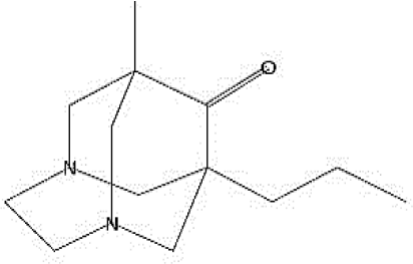
**C_10_H_12_N_6_S**	248.0	5-Methyl-3-(3-propyl)-[1,2,4] triazolo [3,4-b][1,3,4]thiadiazol-6-yl)-1H pyrazole (Heterocyclic)	4.24	248.0	220.0	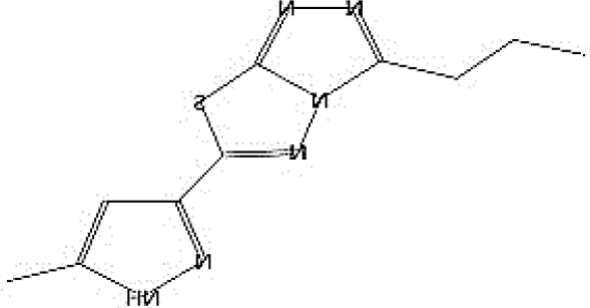
**C_17_H_34_O_2_**	270.0	Metyl hexadecanoate (Sat. fatty acid)	1.67	270.0	87.0 and 74.0	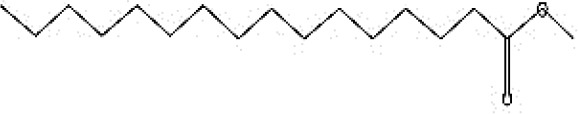
**C_16_H_22_O_4_**	278.0	Dibutyl phthalate (Ester)	0.33	278.0	149.0	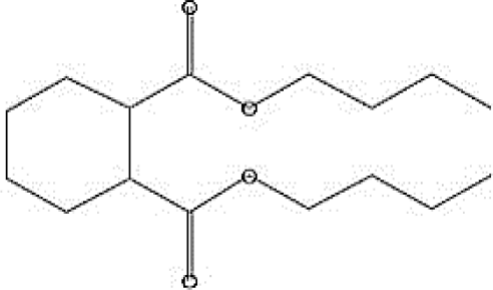
**C_19_H_34_O_2_**	294.0	Methyl linoleate (Unsaturated fatty acid)	3.49	294.0	67.0	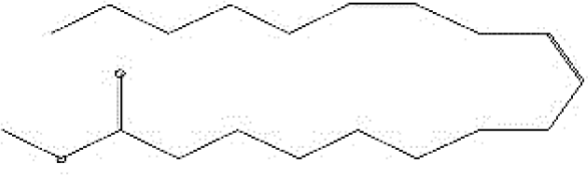
**C_19_H_36_O_2_**	296.0	Methyl-9-octadecenoate (Unsat. fatty acid)	1.39	296.0	55.0	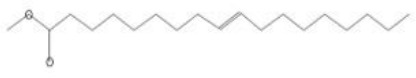
**C_24_H_48_N_2_O_4_**	428.0	n-Heptyl-2-[4-(2(heptyl (methyl) amino]-2-oxoethoxy) butoxy)-N-methylacetamide (Amides)	0.53	428.0	57.0	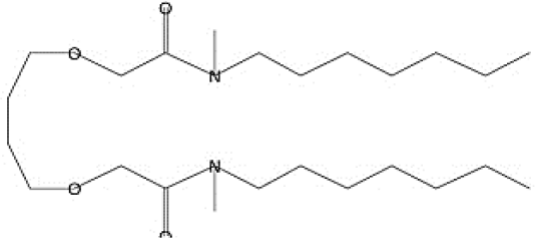
**C_23_H_38_O_2_**	346.0	Methyl 6,9,12.15-doco satetraenoate (Unsaturated fatty ester)	0.58	346.0	41.0	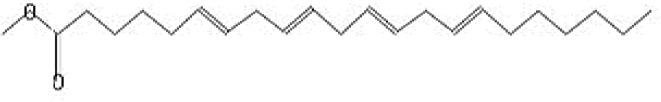
**C_20_H_23_O_2_**	304.0	Arachidonic acid (Unsaturated fatty acid)	1.85	304.0	79.0	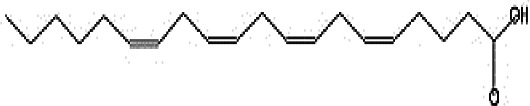
**C_29_H_52_O_2_**	432.0	Isopropyl 5,9,17-hexcosatrienoate (Unsaturated fatty ester)	8.83	432.0	81.0	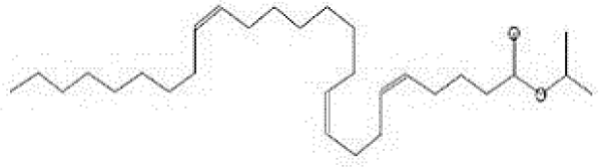
**C_21_H_38_O_2_**	322.0	Methyl 11.14-eicosacienoate. (Unsaturated fatty ester)	1.87	322.0	81.0 and 67.0	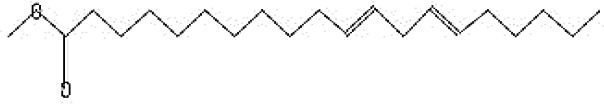
**C_24_H_38_O_4_**	390.0	Bis(2-ethylhexyl) phthalate (Esters)	2.02	390.0	149.0	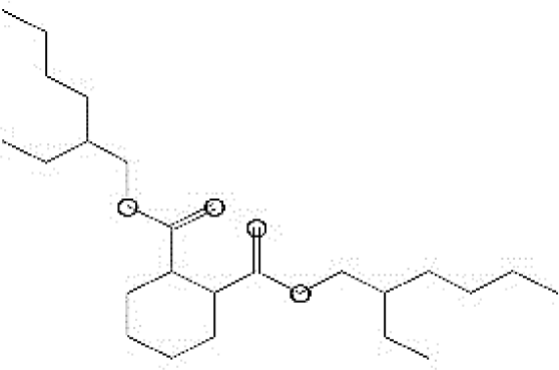
**C_22_H_43_NO**	337.0	Erucylamide or 13-docosenamide (Amide)	1.15	337.0	59.0	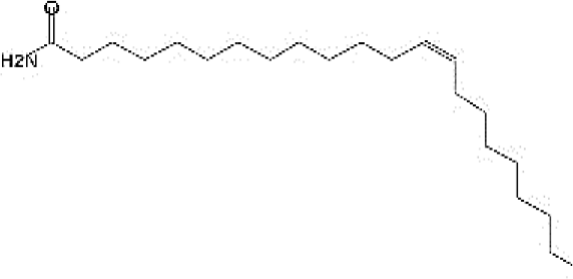
**C_35_H_46_O_2_**	498.0	9(11)-dehydroergosteryl benzoate (Cholesterol)	0.87	498.0	376.0 and 69.0	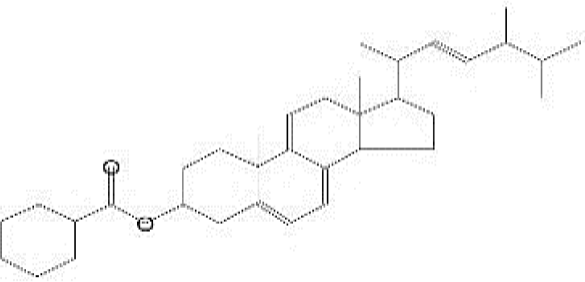

#### 3.3.2 TLC, flash chromatography, and HPLC analysis of the crude extract of *P. chrysogenum*

**Figure 2:**
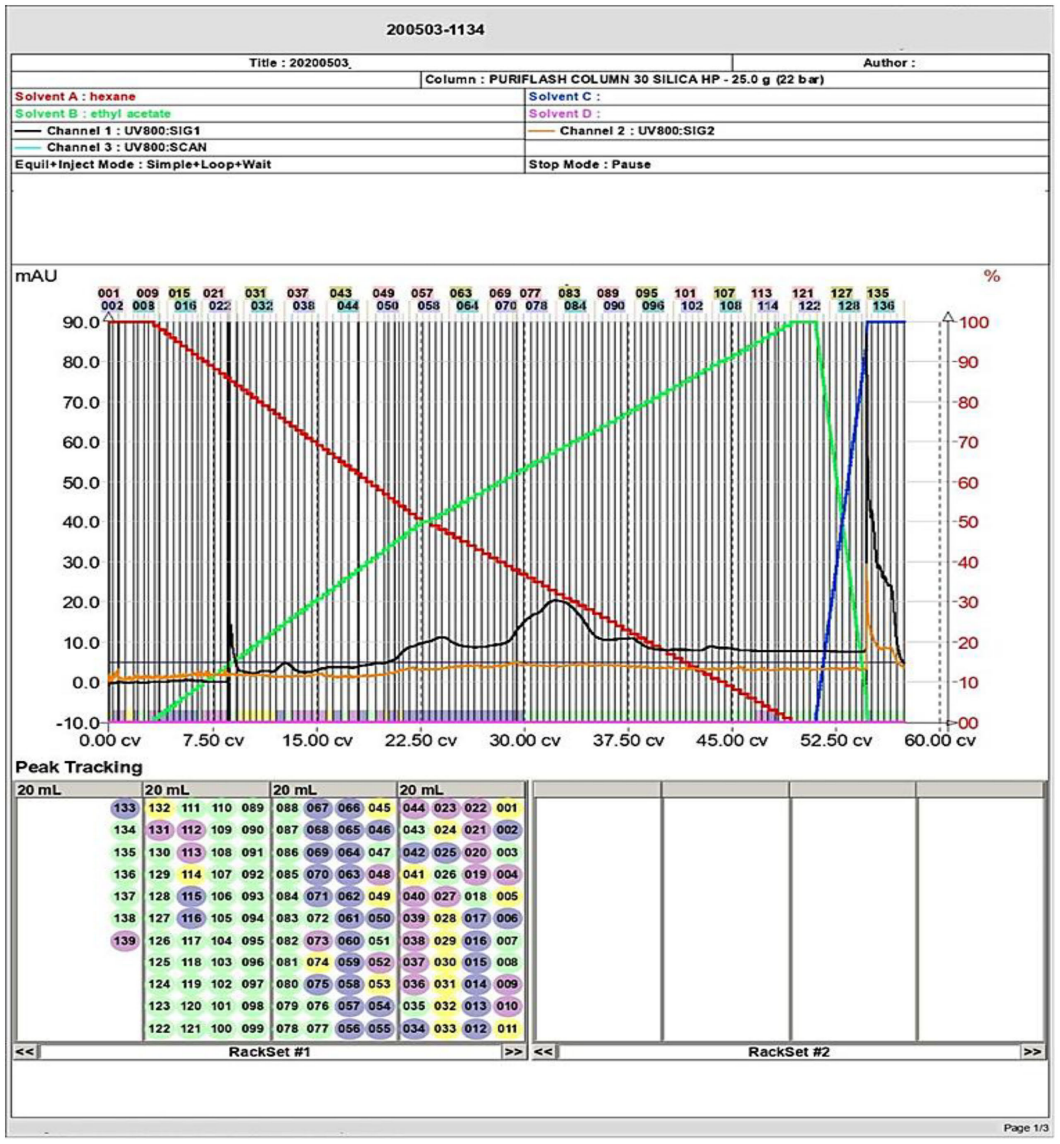
Flash column chromatography analysis of the secondary metabolites and separation of several compounds including the targeted molecule (large peaks) of *P. chyrsogenum*.

The TLC of the extracellular secondary metabolites of *P. chrysogenum* Pc showed the presence of several bands referring to the compounds included within the extract (**Supplementary Figure S1B**). To obtain highly purified bioactive compounds, flash chromatography was performed for the n-hexane extract. As demonstrated in **Figure 2**, the separation had been repeated several times, and there were different peaks in the chromatogram for each of the obtained sub-fractions. However, there were some unique peaks for certain subfractions as illustrated in **Figure 2**.

**Figure 3:**
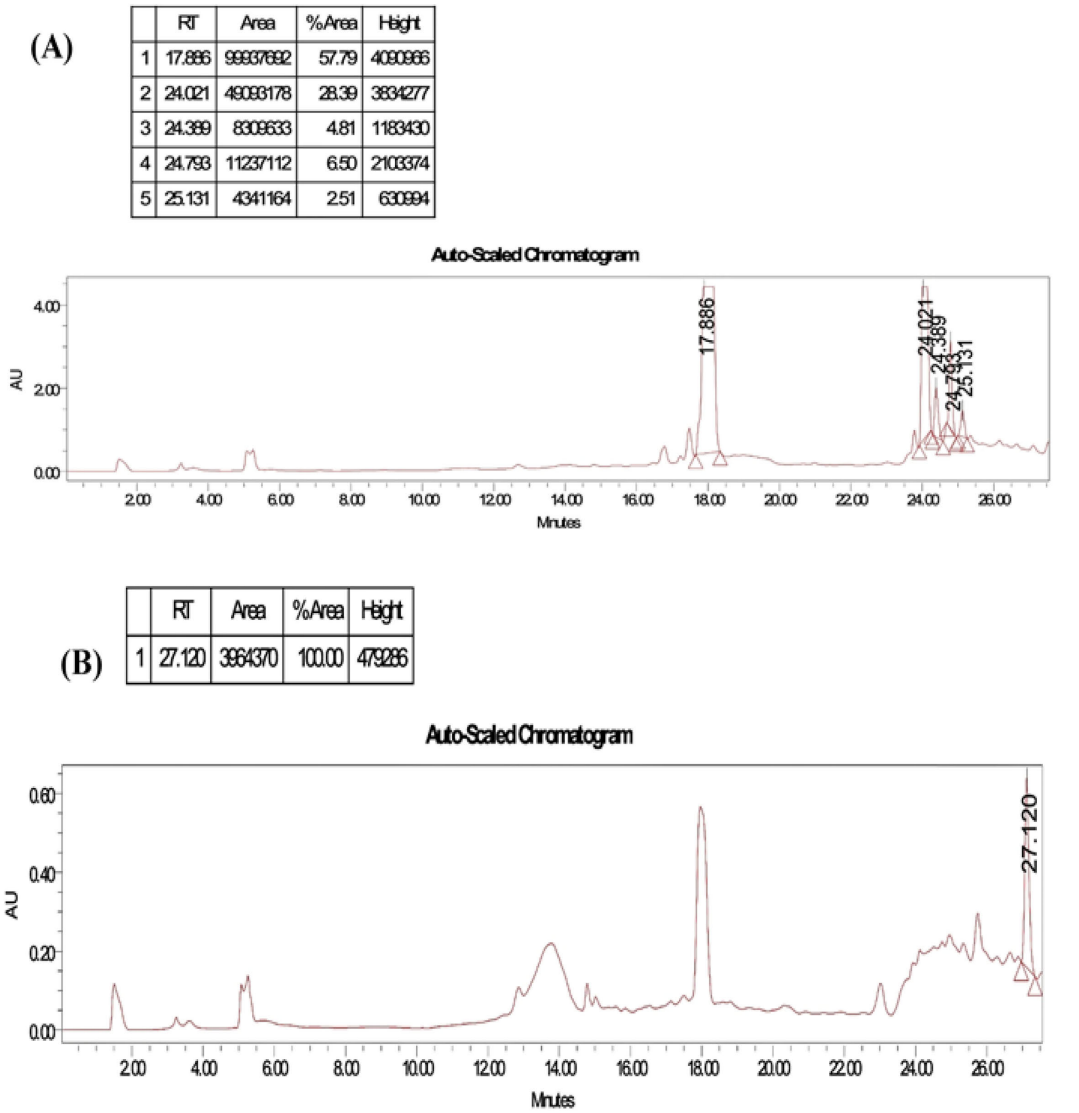
HPLC profiles by running the solvent system for purity test of collected tubes from flash chromatography for *P. chyrsogenums* metabolites. **(A)** Different peaks with a retention time at 17.886, 24.021, 24.399, 24.793, and 25.131 min were detected in different purified compounds. **(B)** A single peak with a retention time at 27.120 min at the last stage of purification.

The purity of the obtained compound in each single sub-fraction of *P. chyrsogenum* was detected through TLC (**Supplementary Figure S1B**). The bioactive compound from the active sub-fraction was then recrystallized by methanol after injection of 1 µg/mL of individual components into HPLC equipment. The analysis displayed a clear peak with a retention time of approximately 17.886 min in the upper region of the curve, and other interfering peaks had retention times of 24.021, 24.399, 24.793, and 25.131 min (**Figure 3A**). Additionally, a single peak with a retention time of 27.120 min appeared at the last stage of purification (**Figure 3B**).

#### 3.3.3 H-NMR and FTIR analyses of the pure fungal compound of *P. chrysogenum*

**Figure 4:**
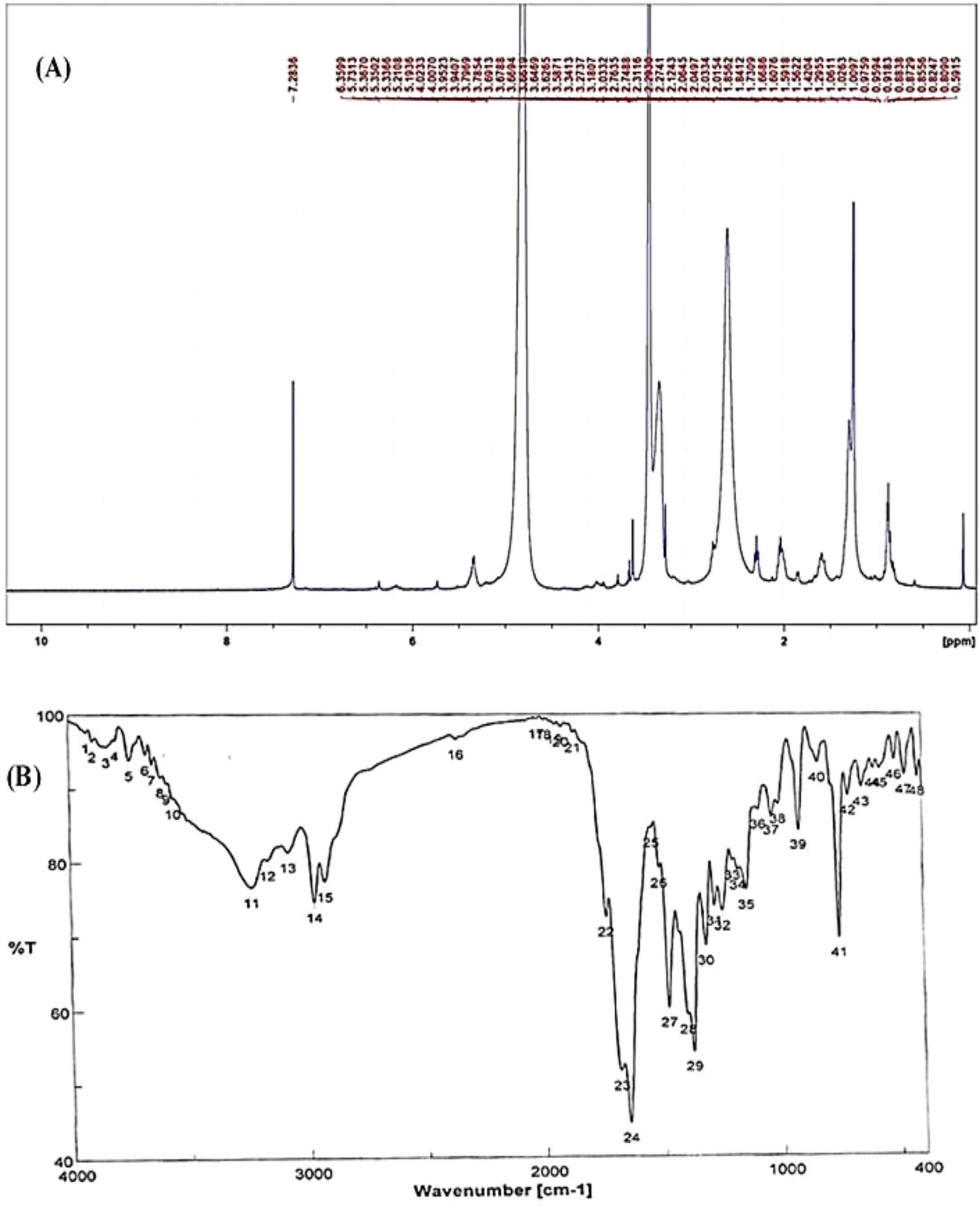
Structural analysis of (Z)-13-docosenamide. **(A)** H-NMR and **(B)** FTIR.

The chemical structure of the pure fungal metabolite of *P. chrysogenum* Pc after HPLC was verified using ^1^H-NMR and FTIR analyses. The resolved ^1^H-NMR signals were divided among 0.94 and 8.0 ppm (**Figure 4A**). ^1^H-NMR (DMSO.d_6_): δ=0.83 (t, 3H, CH_3_), 1.29 (m, 28H, 14CH_2_), 1.56 (m, 2H, CH_2_CH_2_CO), 2.03 (m, 4H, CH_2_-CH=CH-CH_2_), 2.31 (t, 2H, CH_2_CO), 5.33 (2H, -CH=CH-), and 7.28 ppm (S, 2H, NH_2_, exchange with D_2_O).

**Figure 5:**
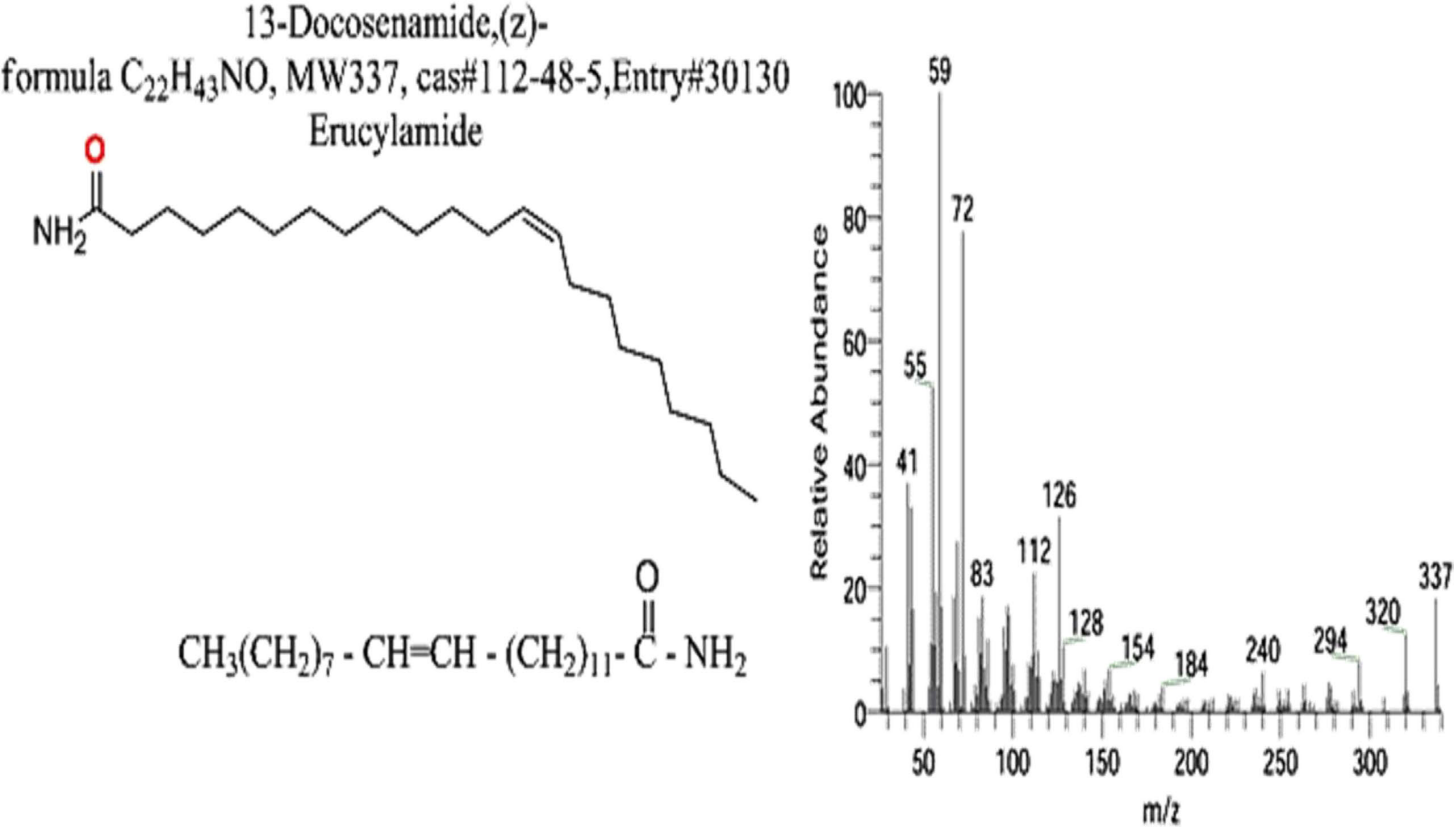
Mass spectrum with structural formula of the purified (Z)-13-docosenamide (erucylamide).

The FTIR spectral analysis of the pure metabolite showed several bands indicating the functional groups (**Figure 4B**). Bands at 3561-3167 cm^-1^ are broad for OH of acid and NH2 groups. While, bands at 1738 and1685 cm^-1^ are for 2C=O ether and ketones groups. Additionally, band at 1650 cm^-1^ is for C=O amide and 1147 cm^-1^ is characterized for (-O-) ether linkage. Hence, IR (KBr) showed bands at 3,561 and 1,650 cm^−1^ for the NH_2_ and C=O amide (**Figure 5**). GC-MS analysis and identification by NMR and FTIR confirm the bioactive compound as (Z)-13-docosenamide or erucylamide with a molecular formula of C_22_H_43_NO and a molecular weight of 337.0 (**Figure 5**). The total ion chromatogram of mass spectroscopic analysis confirmed the purity of the compound (**Figure 5**), with a peak of molecular ion at m/z 337 representing its molecular weight and characteristic fragmentation peaks at m/z 55, 59, 72, and 126 (**Figure 5**).

### 3.4 Biological activity of the purified (Z)-13-docosenamide

#### 3.4.1 Antioxidant activity of the purified (Z)-13-docosenamide

**Figure 6:**
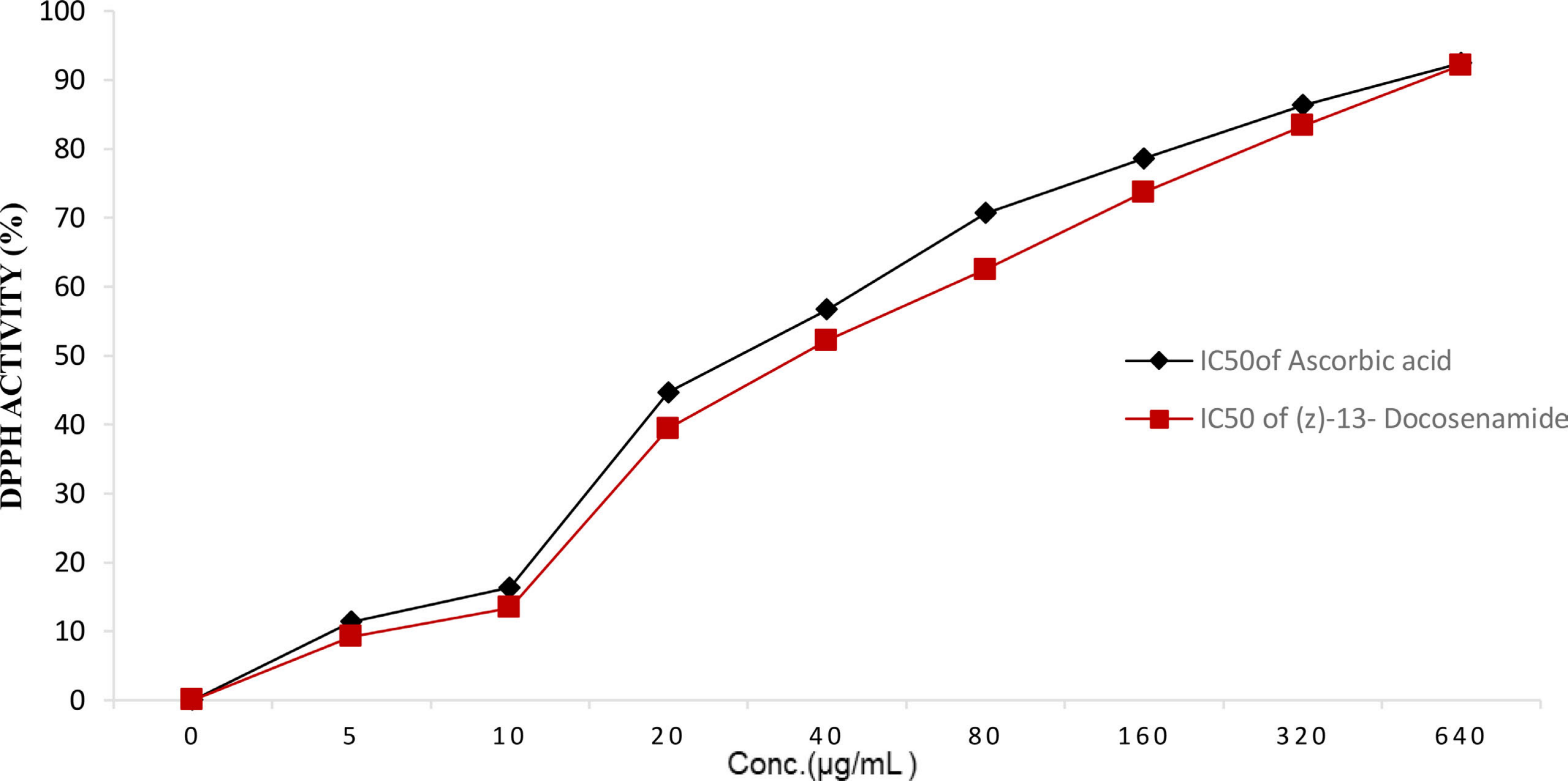
DPPH activity (%) with IC50 = 5 µg/mL for both the purified (Z)-13-Docosenamide and the ascorbic acid.

The purified (Z)-13-docosenamide showed remarkable antioxidant activity as strong as that of the ascorbic acid, estimated as 92.14% and 92.45%, respectively, with IC_50_ = 5 µg/mL for both the (Z)-13-docosenamide and the ascorbic acid (**Figure 6**) (**Supplementary Table S1**).

#### 3.4.2 Antimicrobial activity and MIC of the purified (Z)-13-docosenamide

As shown in **Table 2**, the purified natural (Z)-13-docosenamide possessed antimicrobial activity against *S. aureus, B. subtilis, S. pyogenes, L. monocytogenes, L. innocua, E. coli*, *K. pneumoniae*, *S. typhimurium*, *P. aeruginosa*, *E. cloacae*, *A. fumigatus, P. aurantiogriseum*, and *C. albicans* higher than that of gentamycin (4 µg/mL) and ketoconazole (100 µg/mL) that served as positive controls for bactericidal and fungicidal activity, respectively.

**Table 2:** Antimicrobial activity of the purified (Z)-13-docosenamide compared to gentamycin and ketoconazole as positive controls.

Sample code Tested microorganisms		(Z)-13-docosenamide		Control	
		Mean IZ	± S.D	Mean IZ	± S.D
Gram-positive bacteria	*Staphylococcus aureus ATCC 25923*			*Gentamycin*	
		39.23	0.32	24.01	0.01
	*Bacillus subtilis RCMB 015 (1) NRRL B-543*	37.33	0.06	26.02	0.03
	*Staphylococcus pyogenes* ATCC 19615	28.74	0.24	25.27	0.25
	*Listeria monocytogenes* LMG 10470	31.57	0.4	24.4	0.34
	*Listeria innocua* LMG11387	38.24	0.25	25.29	0.44
Gram-negative bacteria	*Escherichia coli* ATCC 25922			*Gentamycin*	
		38.43	0.08	29.90	0.01
	*Klebsiella pneumoniae* RCMB003(1)ATCC13883	39.77	0.06	27.03	0.06
	*Salmonella typhimurium* RCHB006 (1) ATCC14028	33.33	0.4	22.33	0.2
	*Pseudomonas aeruginosa* LMG 8029	30.9	0.66	20.1	0.09
	*Enterobacter cloacae* RCMB 001 ATCC23355	37.63	0.15	26.36	0.39
Fungi	*Aspergillus fumigatus (RCMB 002008)*	* *		*Ketoconazole*	
		33.37	0.25	17.01	0.02
	*Penicillium aurantiogriseum IMI89372*	36.50	0.12	20.03	0.04
	*Candida albicans RCMB 005003 ATCC 10231*	29.43	0.78	17.9	0.36

The test was done using the diffusion agar technique, well diameter: 6.0 mm (100 µL was tested), RCMB, Regional Center for Mycology and Biotechnology; *NA: No activity. Tested at concentrated samples.

Data represented as mean ± standard deviation (SD).

Positive control for fungi: ketoconazole 100 µg/mL.

Positive control for +ve and –ve bacteria: gentamycin 4 µg/mL.

The MIC of (Z)-13-docosenamide was determined as demonstrated in **Table 3**; the least concentration of (Z)-13-docosenamide that was able to show visible inhibition against the tested bacterial strains was 10 µg/mL, and 20 µg/mL against the tested fungi.

**Table 3:** The antimicrobial activity of different concentrations of (Z)-13-docosenamide for MIC determination.

(Z)-13-docosenamide concentration (µg/mL)	Bacterial strains				Fungal strains	
	*S. aureus*	*B. subtilis*	*E. coli*	*K. pneumoniae*	*A. fumigatus*	*P. aurantiogriseum*
**5**	0.00 ± 0.0	0.00 ± 0.0	0.00 ± 0.0	0.00 ± 0.0	0.00 ± 0.0	0.00 ± 0.0
**10**	8.60 ± 0.40^a^	8.10 ± 0.17 ^b^	10.03 ± 0.03 a^,b,c*^	7.90 ± 0.10 a^,c,d^	0.00 ± 0.0 a^,b,c,d,e^	0.00 ± 0.00 a^,b,c,d^
**20**	15.02 ± 0.01^a^	16.27 ± 0.25 a^,b^	20.03 ± 0.06 a^,b,c*^	12.04 ± 0.04 a^,b,c,d^	18.07 ± 0.12 a^,b,c,d,e^	9.17 ± 0.15 a^,b,c,d,e^
**30**	20.02 ± 0.02 a	25.53 ± 0.06 a^,b^	23.04 ± 0.05 a^,b,c*^	18.14 ± 0.22 a^,b,c,d^	22.60 ± 0.20 a^,b,c,d,e^	17.60 ± 0.10 a^,b,c,d,e^
**40**	28.10 ± 0.10 ^a^	29.27 ± 0.15 a^,b^	25.40 ± 0.10 a^,b,c^	27.33 ± 0.21 a^,b,c,d^	29.37 ± 0.15 a^,c,d,e*^	22.67 ± 0.15 a^,b,c,d,e^
**50**	33.47 ± 0.15 ^a^	34.37 ± 0.32 a^,b*^	27.63 ± 0.11 a^,b,c^	32.33 ± 0.21 a^,b,c,d^	34.04 ± 0.06 a^,c,d,e^	26.30 ± 0.26 a^,b,c,d,e^
**70**	35.23 ± 0.15 ^a^	36.02 ± 0.02 a^,b^	33.17 ± 0.29 a^,b,c^	36.63 ± 0.12 a^,b,c,d*^	36.27 ± 0.15 a^,c,e^	29.37 ± 0.32 a^,b,c,d,e^
**100**	39.37 ± 0.32^a^	39.93 ± 0.06 a^,b*^	35.08 ± 0.08 a^,b,c^	39.77 ± 0.06^c,d^	38.23 ± 0.25 a^,b,c,d,e^	31.13 ± 0.12 a^,b,c,d,e^

*Indicates highly significant differences among the fungal strains according to multiple pairwise comparisons using a one-way ANOVA test at *p* < 0.05.

a–e indicate significant differences at the *p* < 0.05 level for multiple pairwise comparisons among fungal strains using Tukey *post-hoc* adjustment.

Highlighted cells refer to MICs and the resulting inhibition zones.

#### 3.4.3 TEM of the (Z)-13-docosenamide effect on the ultrastructure of *K. pneumoniae* and *P. aurantiogriseum*

**Figure 7:**
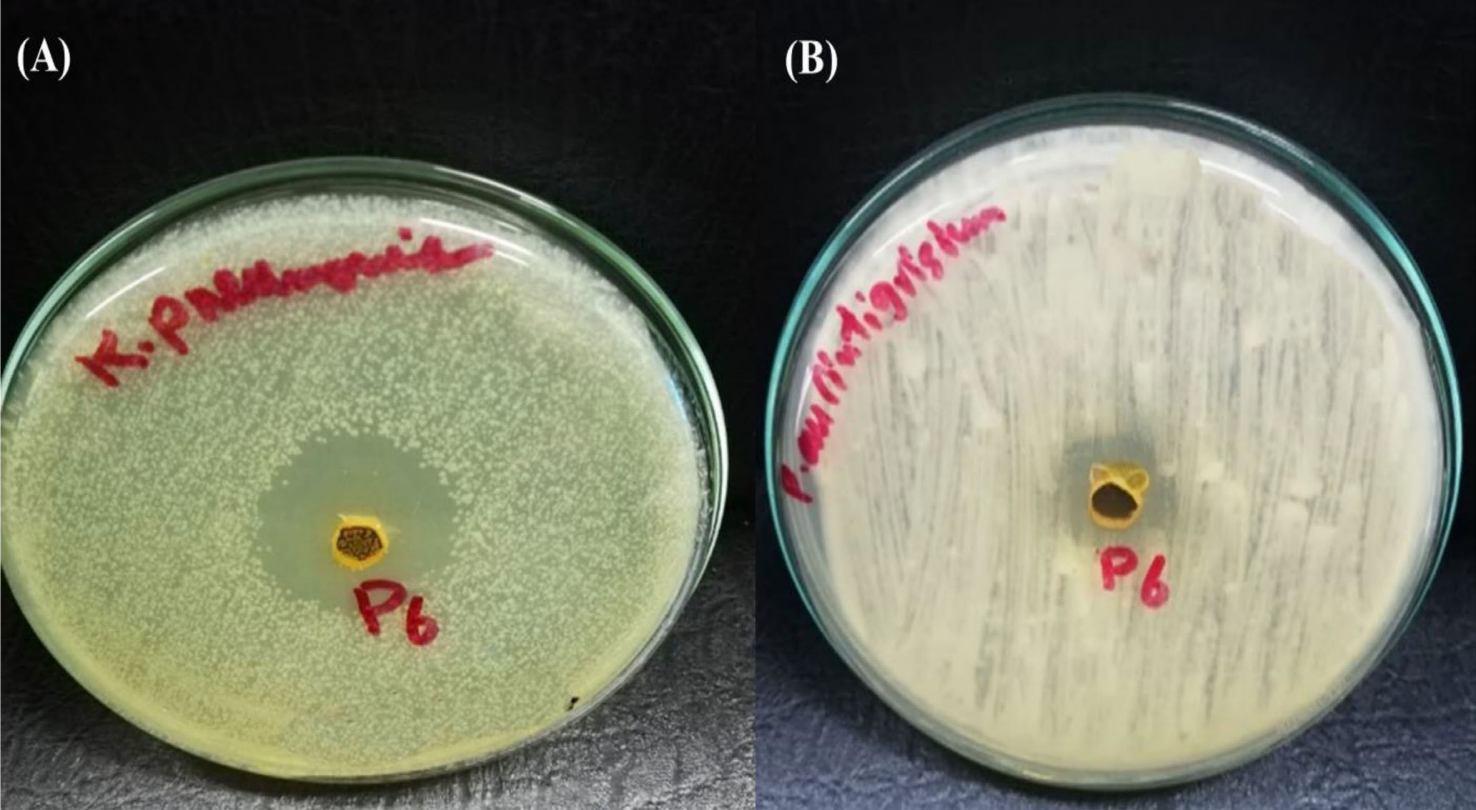
Inhibition zone resulting from the action of purified (z)-13- Docosenamide on Klebsiella pneumonia **(A)**, and penicillium aurantiogriseum **(B)**.

**Figure 8:**
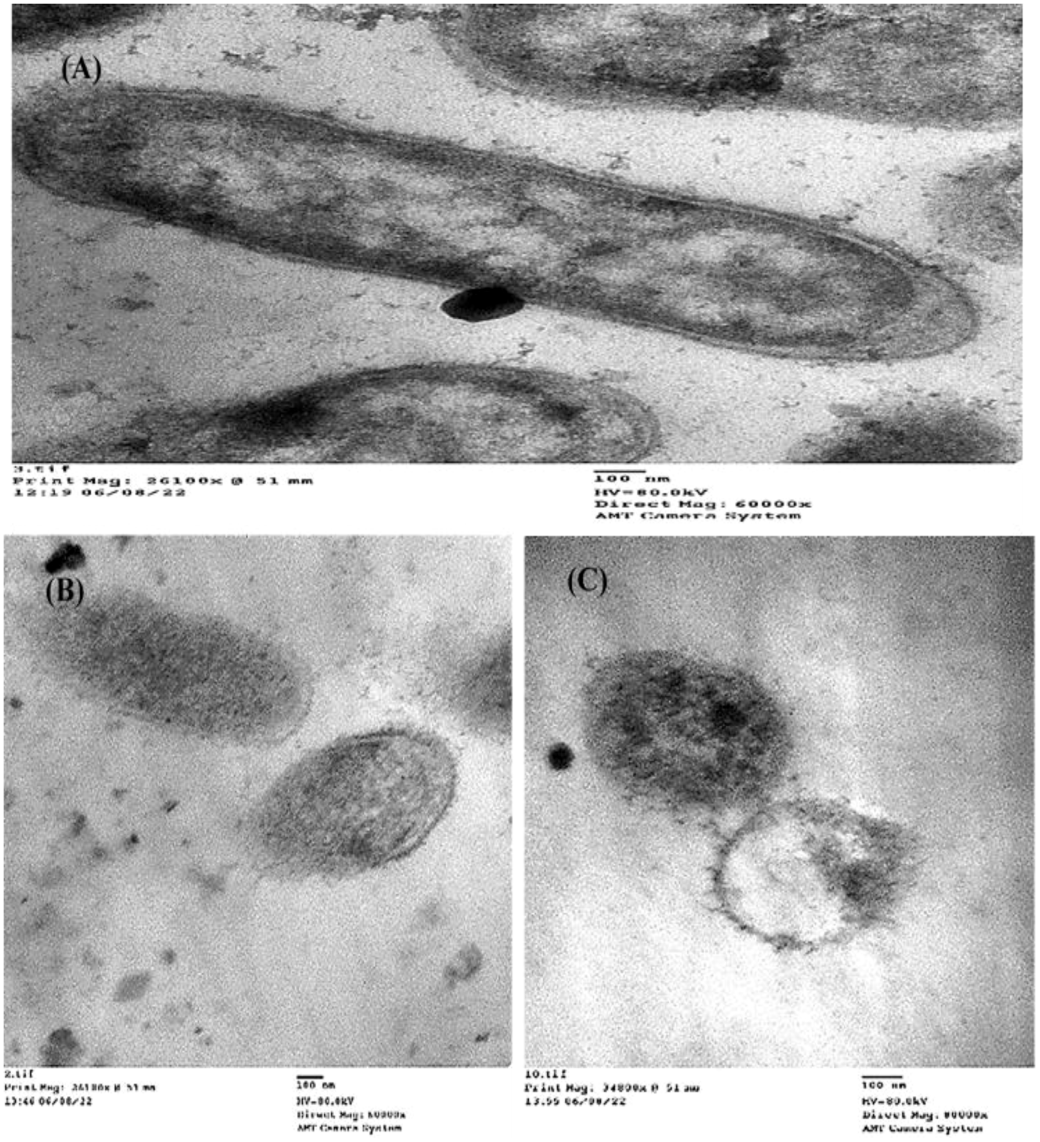
TEM examination of the inhibitory action of (Z)-13-docosenamide on the ultrastructure of *Klebsiella pneumoniae*. **(A)** The ultrastructure of the untreated *K. pneumoniae* and **(B, C)** two shots showing the cell membrane damage of *K. pneumoniae* cells treated with (Z)-13-docosenamide. Micrographs were taken on a magnification of 100 nm.

To detect the effect of the MIC of (Z)-13-docosenamide on the ultrastructure of *K. pneumoniae* and *P. aurantiogriseum* (**Figure 7**), TEM examination was performed. The TEM micrographs (**Figure 8**) show remarkable disruption of the cell membrane of *K. pneumoniae* and leakage of cytoplasmic components in the (Z)-13-docosenamide-treated cells compared to the untreated ones.

**Figure 9:**
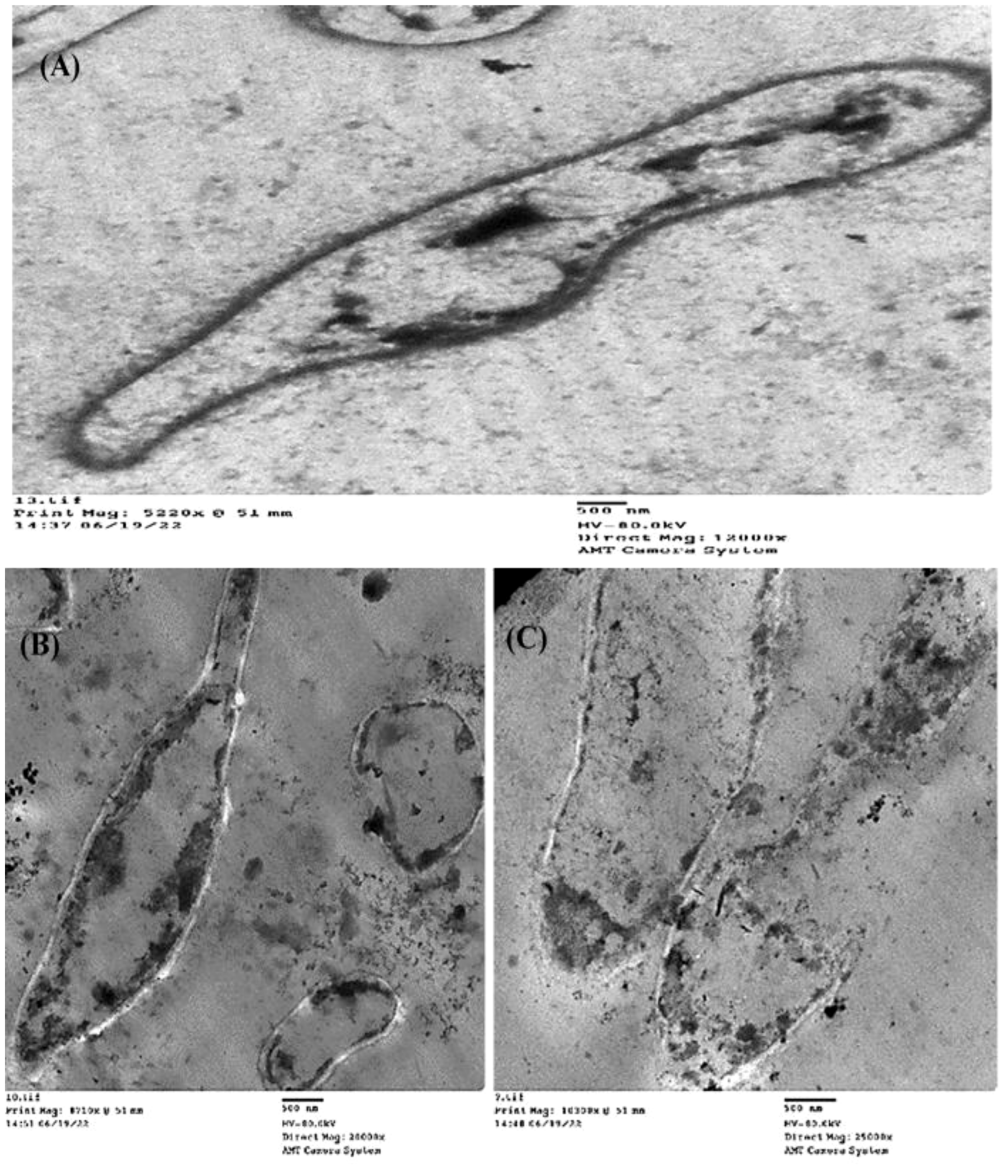
TEM examination of the inhibitory action of (Z)-13-docosenamide on the ultrastructure of *Penicillium aurantiogriseum*. **(A)** The ultrastructure of untreated *Penicillium aurantiogriseum* and **(B, C)** several shots showing the cell wall disruption and leakage of intracellular components of *Penicillium aurantiogriseum* cells treated with (Z)-13-docosenamide. Micrographs were taken on 500 nm.

On the other side, cell shrinkage, disruption of cell wall, and bare central zones (which were formerly occupied by the nucleoid) were noted in the TEM micrographs of (Z)-13-docosenamide-treated *P. aurantiogriseum* cells (**Figure 9**).

#### 3.4.4 Anticancer activity of the purified (Z)-13-docosenamide

**Figure 10:**
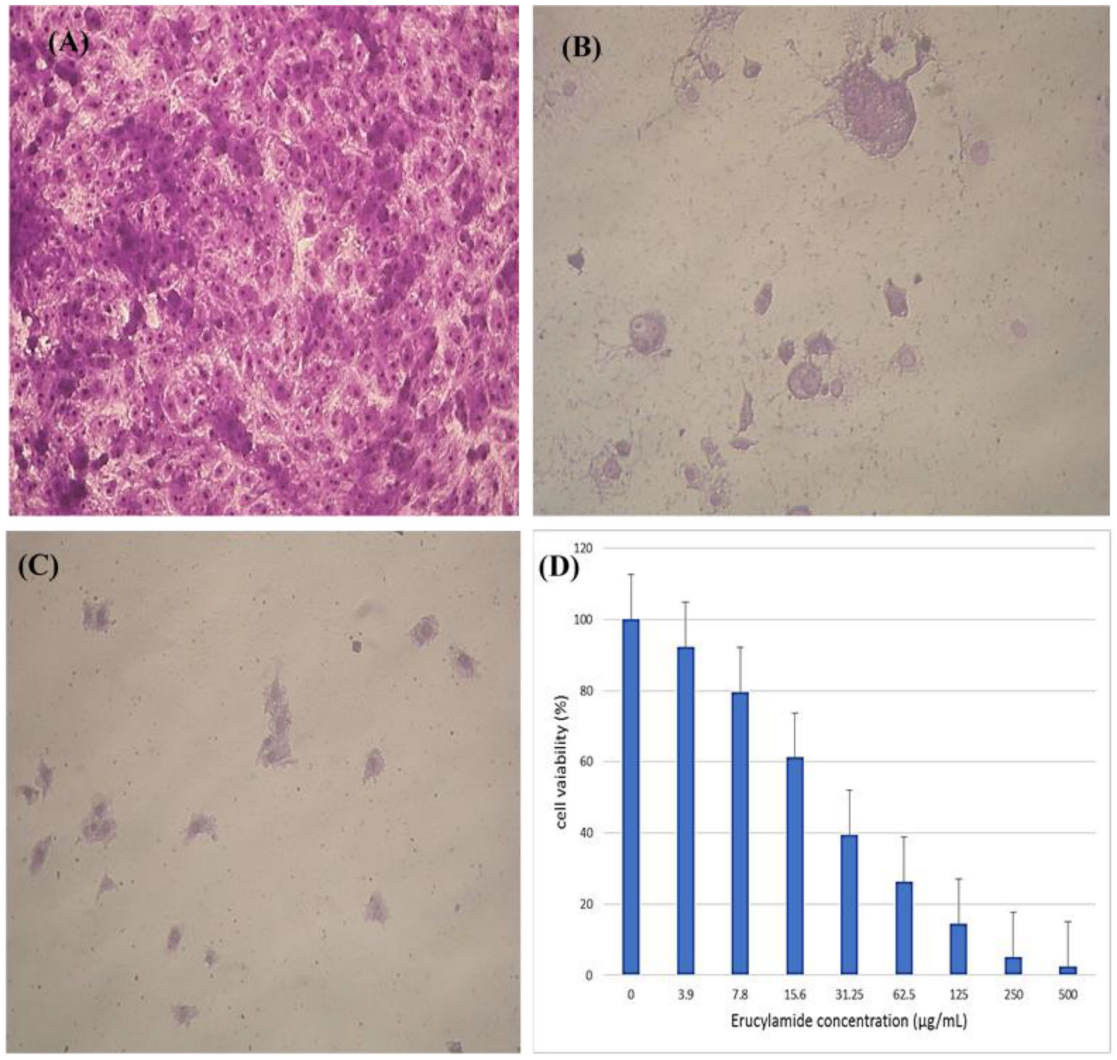
Inhibitory effect of different concentrations of purified (Z)-13-docosenamide on HepG cells. **(A) **Untreated HepG cells. **(B)** HepG cells treated with (Z)-13-docosenamide 100 μg/mL and **(C)** HepG cells treated with (Z)-13-docosenamide 500 μg/mL. **(D)** Inhibitory activities of different concentrations of (Z)-13-docosenamide against the hepatocellular carcinoma cell line HepG2.

**Figure 11:**
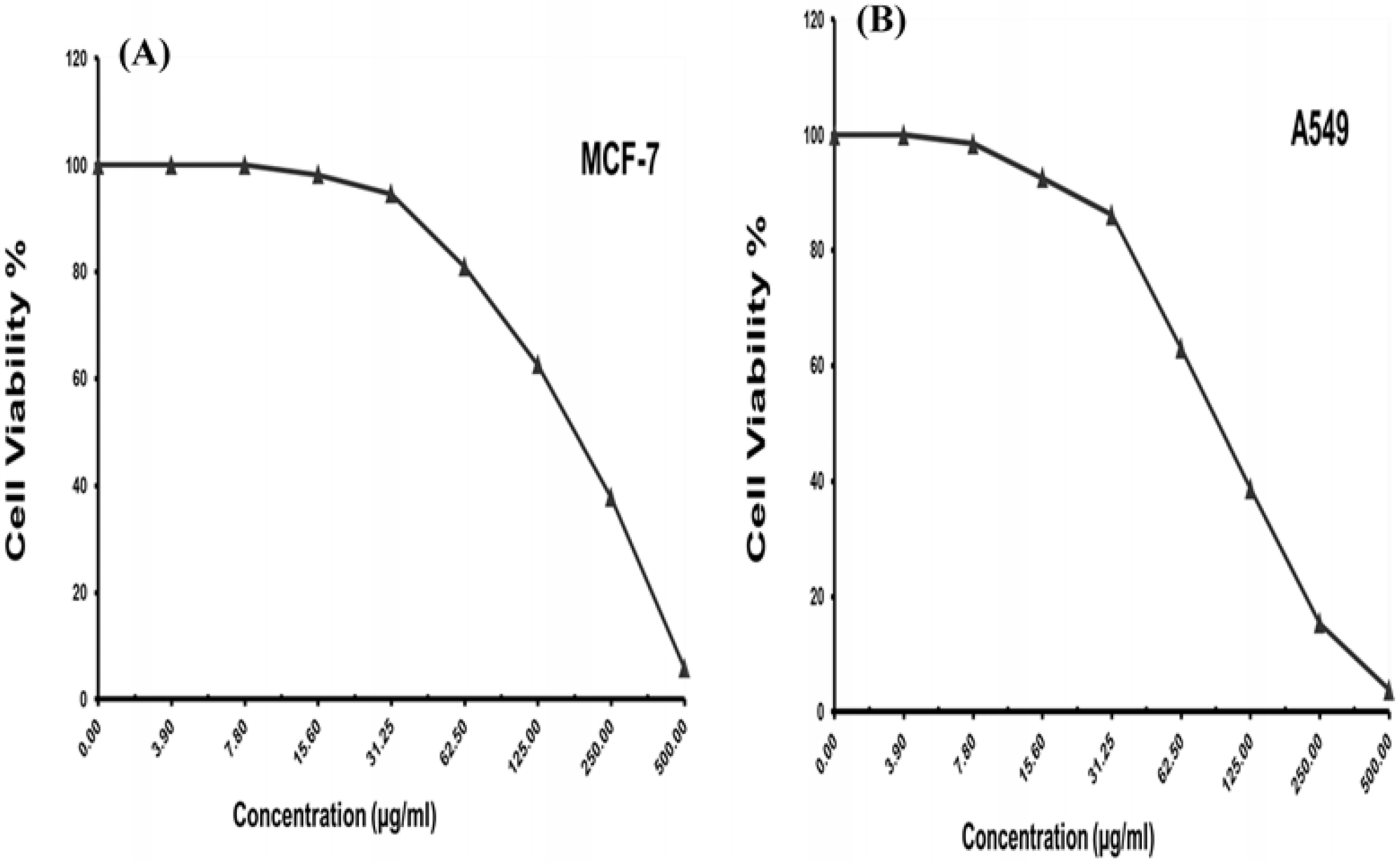
Cell viability and different concentrations of purified (Z)-13-docosenamide against different carcinoma cell lines. **(A)** Breast carcinoma cells (MCF-7). **(B)** Lung carcinoma cells (A-549).

**Figure 12:**
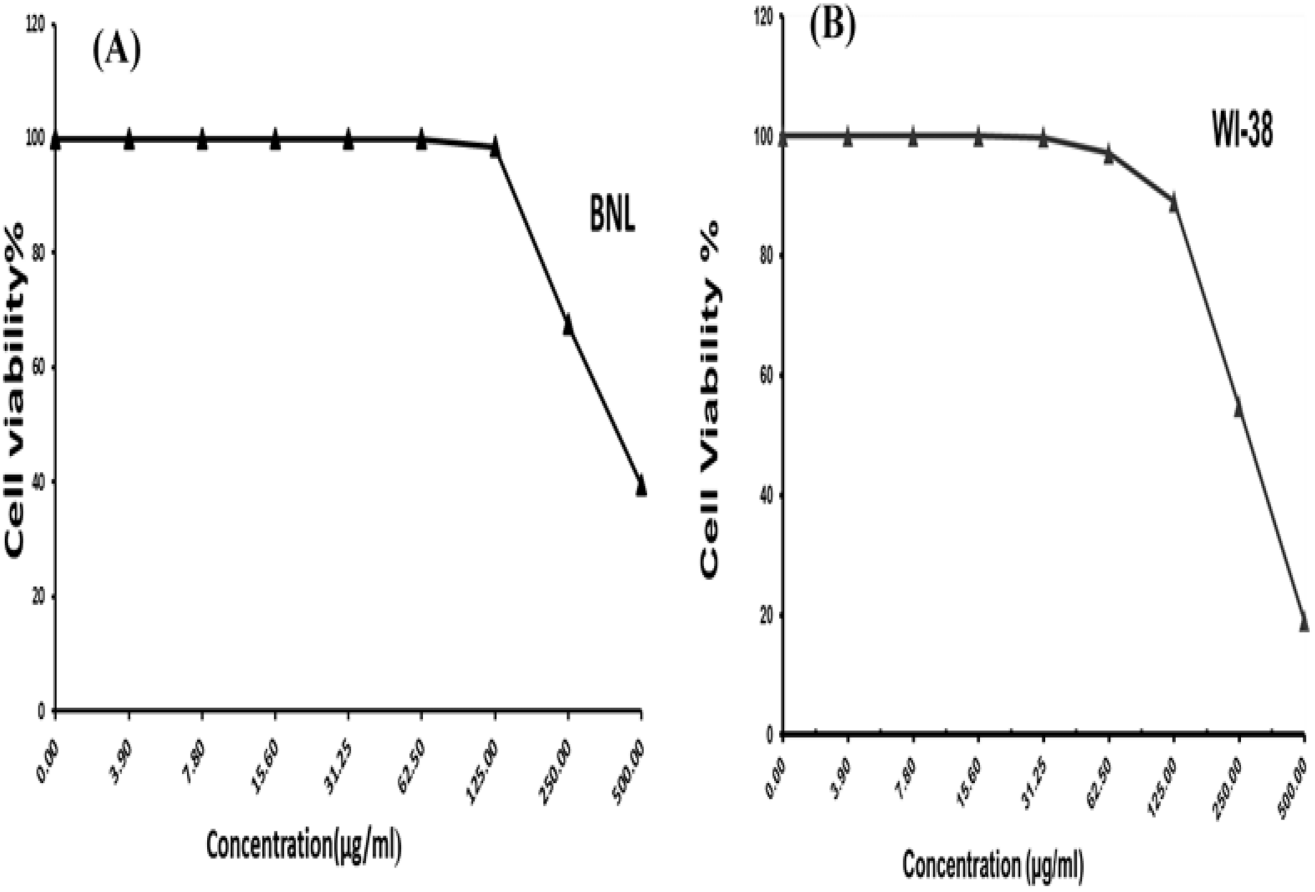
Cell viability percentages at different concentrations of docosenamide on normal cell lines. **(A)** Inhibitory activity against mouse liver normal cells (BNL). **(B)** Inhibitory activity against human lung fibroblast normal cells (WI-38).

The colorimetric MTT test was slightly modified for assessment. The cytotoxic effects of various concentrations of purified (Z)-13-docosenamide on the proliferation of hepatocellular carcinoma cells (HepG2) were determined. The images of the stained cells (**Figures 10A–C**) show the ability of (Z)-13-docosenamide to inhibit the proliferation of HepG cells, which illustrate decreases in stained cells with an increase in (Z)-13-docosenamide concentration. The control HepG-2 cells showed irregular confluent aggregates with a rounded and polygonal cell shape (**Figure 10A**). On the other hand, treatment of the cells with (Z)-13-docosenamide (100 and 500 µg/mL) for 48 h resulted in the shrinkage of polygonal cells and a change to a spherical shape (**Figures 10B, C**). It was also noted that the inhibitory action of (Z)-13-docosenamide against HepG increases with the increase of its concentration with IC_50_ at 23.8 ± 0.8 µg/mL (**Figure 10D**). In addition, the inhibitory action of (Z)-13-docosenamide against MCF-7 and A-549 increases with the increase of its concentration. Furthermore, the IC_50_ values for (Z)-13-docosenamide are 188.46 ± 3.28 and 95.89 ± 2.63 µg/mL against MCF-7 and A-549, respectively, as shown in **Figures 11A, B**. A cytotoxicity assay was conducted on the mouse liver normal cells (BNL) and human lung fibroblast normal cells (WI-38) to explore the potential cytotoxic effects of varied concentrations of natural (Z)-13-docosenamide and assess their safety for application. The preliminary results showed that (Z)-13-docosenamide concentrations until 250 μg/mL are non-toxic and safe on cell tissue and the cells remain viable and constant until 125 μg/mL. The cytotoxicity of this compound demonstrated a concentration-dependent pattern, with higher concentrations leading to decreased cell viability (**Figure 12**). Later on, the cytotoxic activities (CC_50_) of (Z)-13-docosenamide against BNL and WI-38 are 399.54 ± 9.63 and 282.77 ± 8.05 µg/mL, respectively (**Figures 12A, B**).

The results demonstrate that (Z)-13-docosenamide exhibited a substantially antioxidant activity using ABTS radical scavenging with higher IC_50_ values compared with that of ascorbic acid (10.83 ± 0.61 and 3.46 ± 0.34 µg/mL, respectively). This confirms that they have low antioxidant activity using the ABTS indicator, which favors the creation of oxidative stress in tumor cells (**Table 4**).

**Table 4:** Evaluation of the antioxidant activity of (Z)-13-docosenamide using ABTS radical scavenging.

Sample concentration (µg/mL)	(Z)-13-docosenamide		Ascorbic acid standard	
	ABTS scavenging %	S.D. (±)	ABTS scavenging %	S.D. (±)
1,000	96.71	0.83	98.04	0.28
500	90.43	1.29	96.71	0.57
250	85.68	1.06	94.56	0.62
125	81.27	0.71	90.42	0.94
62.5	76.49	0.65	86.73	0.31
31.25	69.08	1.24	79.68	0.63
15.6	56.42	0.96	74.68	0.84
7.8	45.93	1.45	69.23	0.69
3.9	37.82	1.04	54.02	0.85
2	30.91	0.72	39.54	1.28
1	24.43	0.39	32.78	1.64
0.5	19.80	0.56	28.97	0.49
0	0	0	0	0

In the present investigation, we assessed the activities of many free radical enzymes in HEPG-2 cells treated with (Z)-13-docosenamide, including MDA, SOD, CAT, and GSH (**Table 5**). Additionally, the amount of total protein was assessed as a measure of the compound’s cytotoxic effect. The findings show that treating the HEPG-2 cells with the compound’s IC_50_ value increased the level of MDA, SOD, and CAT while simultaneously decreasing CAT in comparison to the control cells. Furthermore, the ROS levels increased compared to the control cells. These findings unequivocally demonstrate that (Z)-13-docosenamide’s lethal action is largely caused by upsetting the equilibrium between the antioxidant system and the generation of free radicals, or ROS. This is accomplished by raising ROS levels, as shown by the notable rise in SOD levels, which initiate ROS production (**Table 6**).

**Table 5:** Effect of (Z)-13-docosenamide on oxidative enzymes by the ELISA technique.

Sample code Oxidative enzymes (nmol/mg protein)	(Z)-13-docosenamide	Cell control (non-treated)
Catalase	8.34 ± 0.08	14.11 ± 0.22
Superoxide dismutase (SOD)	55.54 ± 1.89	36.51 ± 1.60
Glutathione (GSH)	6.25 ± 0.28	3.79 ± 0.13
Malonaldehyde (MDA)	2.70 ± 0.13	0.71 ± 0.06

**Table 6:** Evaluation of the mechanism of cytotoxicity of (Z)-13-docosenamide against the HEPG2 cell line, ROS determination using ELISA.

Sample code	Tested concentration (µg/mL)	ROS (pg/mL)	% of control
(Z)-13-docosenamide (treated cells)	IC_50_ = 23.8	85.75 ± 1.14	127.84
HEPG2 cells (control)	0	67.09 ± 1.63	100

### 3.5 Molecular docking analysis and binding energetics for (Z)-13-docosenamide

The molecular docking analysis revealed distinct binding affinities of the purified compound (Z)-13-docosenamide for each tested protein complex; TGF-β II demonstrated a binding energy of −3.3 kcal/mol, OMP-A showed −5.5 kcal/mol, and tubulin beta chain exhibited −6.1 kcal/mol. Notably, no hydrogen bonds were observed in any of the complexes, suggesting that the binding mechanisms are predominantly driven by hydrophobic interactions. These findings provide valuable insights into the nature of the molecular recognition processes between (Z)-13-docosenamide and these therapeutically relevant protein targets. The results (**Table 7**) summarize the docking scores and hydrogen bonding characteristics observed for each protein–ligand complex. The binding energies ranged from −3.3 to −6.1 kcal/mol, indicating moderate to strong binding interactions across the different protein targets.

**Table 7:** Docking scores and hydrogen bonding analysis of (Z)-13-docosenamide with TGF-β II, OMP-A, and Tubulin beta chain proteins at 298.15 K.

Ligand	TGF-β II	OMP-A	Tubulin beta chain
(Z)-13-docosenamide	−3.3 kcal/mol, no hydrogen bonds	−5.5 kcal/mol, no hydrogen bonds	−6.1 kcal/mol, no hydrogen bonds

**Figure 13:**
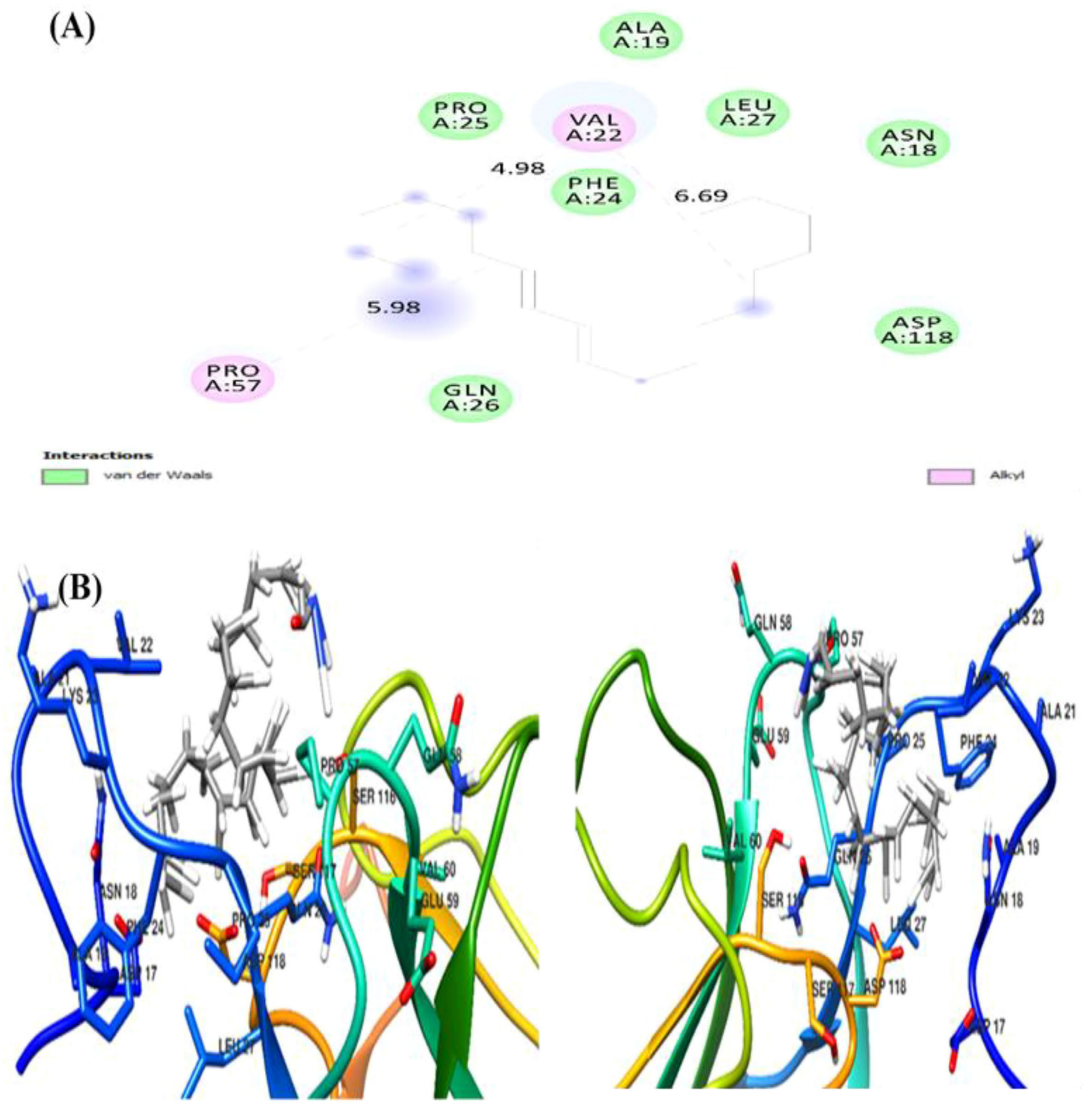
Molecular docking visualization of (Z)-13-docosenamide bound to TGF-β II. **(A)** The 2D interaction diagram highlighting key residues involved in binding. **(B)** The 3D structural representation of the protein–ligand complex. Green circles indicate residues involved in van der Waals interactions, while pink circles represent residues involved in alkyl interactions.

**Figure 14:**
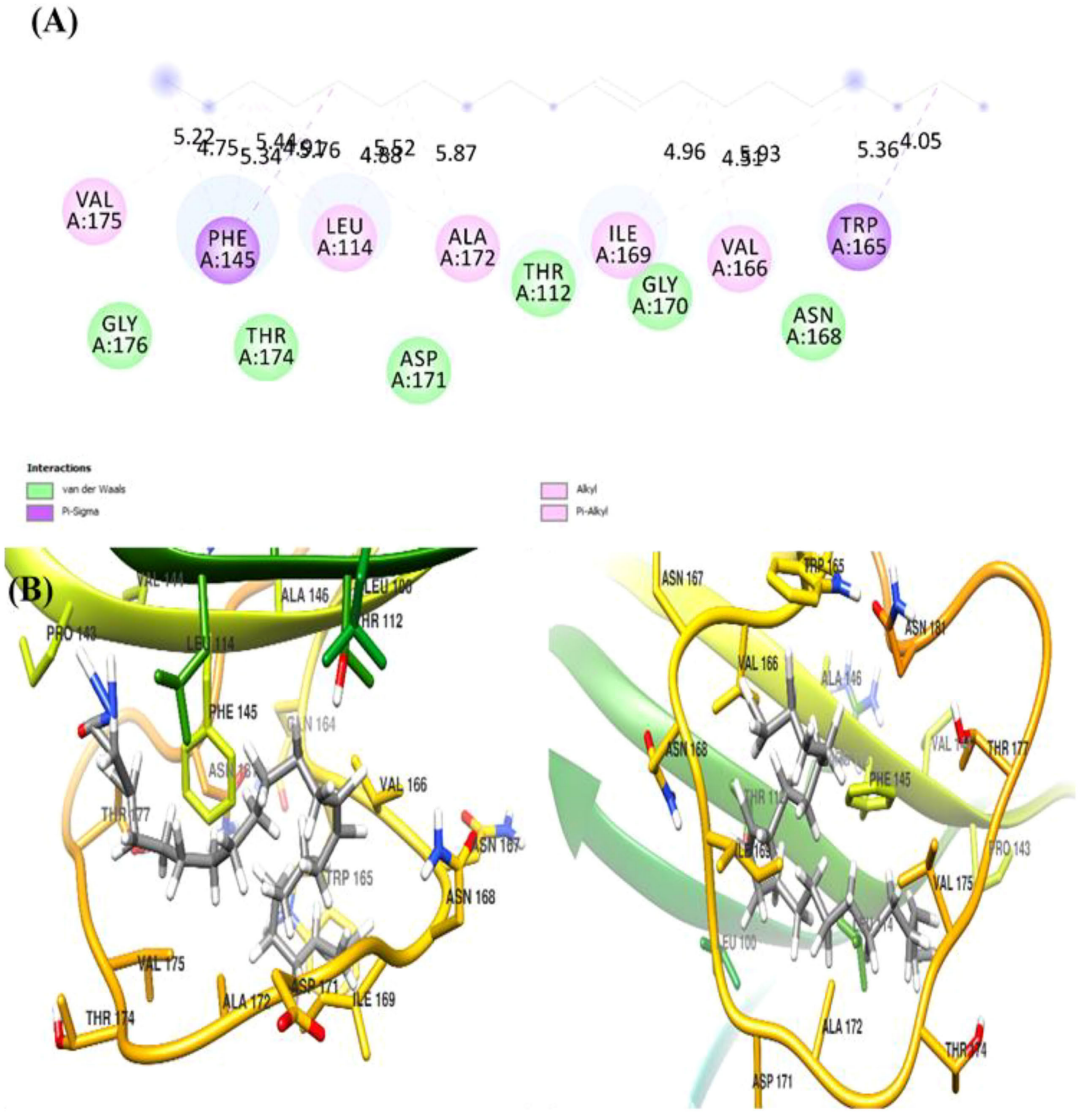
Docking analysis of (Z)-13-docosenamide with OMP-A. **(A)** The figure shows both 2D interaction mapping and 3D structural representation. **(B)** The binding site residues are highlighted with specific interaction types indicated by different colors (green for van der Waals and pink for alkyl interactions). Distance measurements (in Å) between key interaction points are displayed.

**Figure 15:**
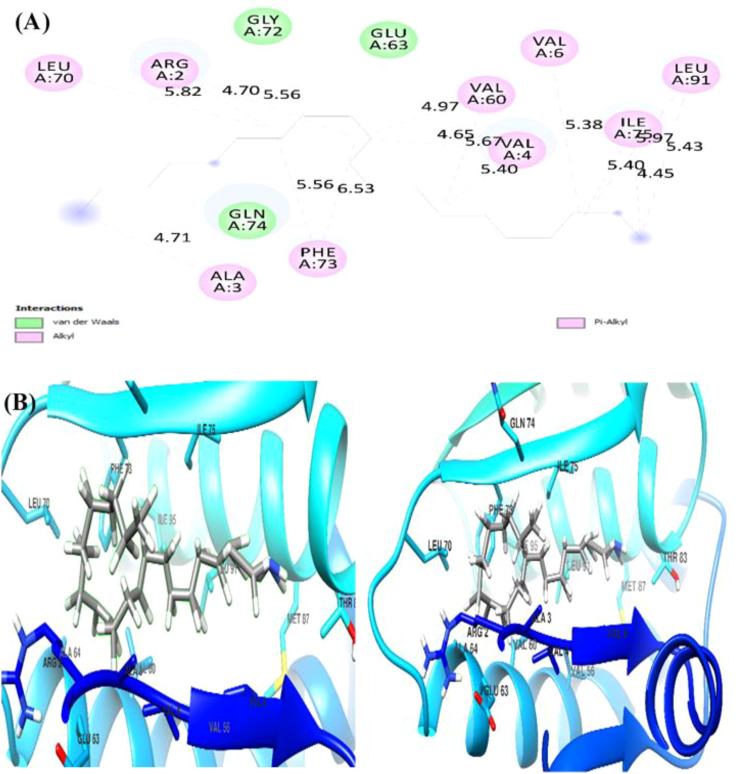
Molecular docking results of (Z)-13-docosenamide with tubulin beta chain protein. **(A)** The figure illustrates both 2D interaction mapping and **(B)** 3D conformational arrangement. Key residues involved in binding are highlighted, with interaction distances marked in Å.

The docking analysis of (Z)-13-docosenamide with TGF-β II revealed specific binding interactions within the protein’s active site. The ligand demonstrated a binding energy of −3.3 kcal/mol, indicating moderate affinity for the receptor. The binding pose analysis identified several key residues involved in the interaction, including ALA.19, PRO.A25, VAL.A22, LEU.A27, and ASN.A18, which form a binding pocket accommodating the ligand (**Figure 13**). In addition, the interaction between (Z)-13-docosenamide and OMP-A exhibited stronger binding characteristics compared to TGF-β II, with a binding energy of −5.5 kcal/mol. The binding site analysis revealed interactions with multiple residues including VAL.A175, PHE.A145, LEU.A114, and TRP.A165. The ligand adopted a conformation that maximized hydrophobic contacts within the binding pocket (**Figure 14**). Furthermore, the strongest binding affinity was observed between (Z)-13-docosenamide and the tubulin beta chain protein, with a binding energy of −6.1 kcal/mol. The binding site analysis revealed interactions with residues LEU.A70, ARG.A2, GLY.A72, GLU.A63, and VAL.A6. The ligand positioned itself in an optimal orientation within the binding pocket, facilitating strong hydrophobic interactions (**Figure 15**).

## 4 Discussion

Recently, natural products from fungi have received more attention as a safe source for several compounds and derivatives with antioxidant, cytotoxic, and antimicrobial properties (Uzma et al., 2018; Abdel-Shafi et al., 2020; Enan et al., 2020). Filamentous fungi are recognized for their ability to generate secondary metabolites that are safe and useful in a range of applications (Samuel et al., 2011). Ascomycota members are able to produce diverse products with antimicrobial, antioxidant, and anticancer properties (Zain et al., 2014). This study targeted the secondary metabolites of filamentous fungi. In this regard, the secondary products of all fungal isolates were examined for their antioxidant and antimicrobial efficiency.

The extracts of all fungi possessed antimicrobial and antioxidant activity with varying values when comparing all fungal strains with control gentamycin or ketoconazole as well as pairwise comparison between all fungal strains. It was clear from the findings of significant differences that the extracellular extract of all isolates possessed higher antioxidant, bactericidal, and fungicidal efficiency than the intracellular extract. Among the tested strains, the strain *P. chrysogenum* Pc was selected as the isolate whose extracellular secondary metabolites exhibited the highest antioxidant and antimicrobial activity in the preliminary bioactivity tests as it indicates highly significant differences among the fungal strains and control using multiple comparisons. The bioactivity results of this work revealed the presence of several bioactive chemicals in the metabolites of the fungal isolates tested, in accordance with the study of Al-Saleem et al. (2022). For instance, the DPPH test of the extracellular extract of *P. chrysogenum* Pc showed substantial antioxidant activity, which is statistically significant over the control. The scavenging DPPH value was found to be 93.27 ± 0.38 for the extracellular extract with an IC_50_ at 5 µg/mL compared to the antioxidant efficiency of ascorbic acid at 92.04 ± 0.49, which served as a positive control and is known as the most influential natural antioxidant (Shi et al., 2015; Al-Saleem et al., 2022).

*P. chrysogenum* exhibited potent antimicrobial activity towards *B. subtilis, S. aureus*, *K. pneumoniae*, *E. coli*, *P. aurantiogriseum*, and *A. fumigatus.* Previous studies using various fungal isolates have shown that filamentous fungi can produce antimicrobial metabolic compounds (Octarya et al., 2021). The bioactivity results of this work revealed the presence of several bioactive chemicals in the metabolites of the fungal isolates tested.

Furthermore, the selected strain was identified with 18s rRNA gene amplification, which confirmed the isolate to be *P. chrysogenum* (El-Gazzar et al., 2023). In addition, the extracellular extract of the selected strain underwent several analyses to purify and identify the present bioactive compounds. The GC-MS analysis reveals the presence of 16 variable bioactive compounds in the extracellular secondary metabolites of *P. chrysogenum* Pc. These compounds included heterocyclic compounds, aldehydes, amides, and polyaromatic compounds, which are known for their antimicrobial properties (Octarya et al., 2021). Furthermore, some studies showed that the presence of anhydrides, naphthalene, cholesterols, and phthalates is responsible for possessing antimicrobial and anticancer activity (González-Menéndez et al., 2018). On the other side, the existence of pyrazole and fatty amide such as (Z)-13-docosenamide in the extract provides significant antimicrobial, antioxidant and anticancer properties (Gueule et al., 2015; Naim et al., 2016; Ker et al., 2017; Xie et al., 2021). In addition, the presence of additional helpful compounds such as esters, pyrazine, pyridine, and unsaturated fatty esters, which have antiproliferative characteristics, was observed (Chaban et al., 2019; Peng et al., 2020). Furthermore, the presence of positively charged esters suppresses the microbial cells electrostatically with the loss of their cell viability and suppression of DNA synthesis, which kills the cells (González-Menéndez et al., 2018; Octarya et al., 2021).

After detecting such a large number of bioactive compounds in the GC-MS analysis, it was important to prepare and purify the bioactive compounds. Therefore, flash chromatography was performed using a variety of solvents under short time periods to optimize the separation and purification processes (Uckoo et al., 2011). Furthermore, the Flash column includes an evaporating light-scattering detector, which enables the system to track the substances not absorbing UV and detect non-volatile chemicals. In addition, the use of columns with gradient elution, such as C18 flash cartridges, provided effective separation with rapid extraction of subfractions containing highly purified and the most accurately separated components (Uckoo et al., 2011; Al-Azzawi et al., 2022). Throughout the extraction process, polar (chloroform) and non-polar (n-hexane) solvents form hydrogen bonds with the target material (Abdel-Shafi et al., 2020), which allows obtaining contaminant-free sharp fractions that contain the bioactive substances (Prakasham et al., 2014; El-Sayed et al., 2020).

Furthermore, several analyses were conducted following the approach of Abdel-Shafi et al. (2020) to detect the chemical structure of the bioactive compound in the extracellular secondary metabolites of *P. chrysogenum* Pc. Depending on the NMR and FTIR spectral analysis, the bioactive material was distinguished as a fatty amide derivative identified as (Z)-13-docosenamide or erucylamide. (Z)-13-docosenamide is a therapeutic fatty acid amide, which has been reported to be extracted from plants, animals, and microbes (Kim et al., 2018; Tamilmani et al., 2018; Hamberger and Stenhagen, 2003), and its biological activities against a variety of diseases such as cancer, bacterial infection, parasitic infection, inflammation, diabetes, and obesity have been reported (Li et al., 2017; Xie et al., 2021). It is a nontoxic natural substance found in plants and derived from filamentous fungi and is a strong acetylcholinesterase inhibitor (Mosmann, 1983). In certain investigations, docosenamide has shown antioxidant action. While free radicals are very reactive chemicals that can harm cells and play a role in a number of disorders, docosenamide may help shield cells from oxidative stress that can harm proteins, lipids, and DNA, by scavenging free radicals. ROS are reactive oxygen-containing chemical species that consist of free radicals and non-radical molecules like superoxide and H_2_O_2_, respectively (Hasan et al., 2021). Numerous diseases, including cancer, have oxidative stress caused by ROS as a contributing factor. Many mechanisms that can produce antioxidants to defend against oxidative stress, shield cells from harmful effects, and prevent diseases are a gift of human physiology (Mazumder et al., 2020).

(Z)-13-docosenamide has been shown to be effective against pathogenic microorganisms (Gomha et al., 2015). Similarly, our findings show the purified (Z)-13-docosenamide that has antimicrobial activity with MICs of 10 µg/mL for the tested pathogenic bacteria and 20 µg/mL for the tested fungi compared to the study of Priya and Maneemegalai (2018) that observed the antimicrobial activity of (Z)-13-docosenamide against *K. pneumoniae, Proteus mirabilis, S. pneumoniae*, and *S. aureus* as well as the study of Mahalakshmi et al. (2020) that observed the antifungal activity of (Z)-13-docosenamide. Furthermore, the TEM micrographs herein showed that the antimicrobial efficiency of (Z)-13-docosenamide depends on making pores in the cell wall and cell membrane, which consequently leads to the leakage of intracellular components leading to complete death of *K. pneumoniae* and *P. aurantiogriseum*. In a previous study, (Z)-docosenamide exhibited promising antibacterial action against 60% of human pathogenic microorganisms such as *Salmonella typhi* and *S. paratyphi* (Priya et al., 2018). Additionally, previous studies have reported the presence of a variety of secondary metabolites, including terpenoids, phenols, alkaloids, quaternary alkaloids, coumarins, flavanoids, and steroids, which are attributed to their antifungal properties (Priya and Maneemegalai, 2018; Octarya et al., 2021; Xie et al., 2021). Consequently, a previous investigation found that the most prevalent components in *R. annamalayana* are (Z)-13-docosenamide and 9-octadecenamide. It is logical to believe that these chemicals may be the cause of *R. annamalayana*’s antifungal action (Mahalakshmi et al., 2020), whereas previous studies have shown that the inhibitory potential of fungal products is related to the activity of various chemicals and organic acids (González-Menéndez et al., 2018; Octarya et al., 2021). The positively charged moieties suppress the microbial cell walls elecrostatically with loss and ultimately lysis of cells (Gueule et al., 2015; Xie et al., 2021). They interact with particular molecules, penetrate cell membranes, or stop the development of cell walls or essential components like glycan and chitin. Because of their cationic amino acid residues and capacity to form dimers, lipopolysaccharides and lipoteichoic acid—negatively charged constituents of microbial cell membranes—are broken apart and depolarized.

Furthermore, prior research has shown (Z)-13-docosenamide’s antiproliferative efficacy against some cancer cell lines (Mou et al., 2013; Kim et al., 2015; Karakus et al., 2018). The preliminary results herein showed that (Z)-13-docosenamide concentrations until 250 μg/mL are non-toxic and safe on cell tissue and the cells remain viable and constant until 125 μg/mL. Exceptionally, the (Z)-13-docosenamide concentration was about 10 times higher than the MIC mentioned herein, pointing to the safe use of (Z)-13-docosenamide. The outcomes remained within the safe limits set by the National Cancer Institute of the United States (Lagunes-Castro et al., 2015). Likewise, the MTT assay demonstrated that the (Z)-13-docosenamide extracted from *P. chrysogenum* Pc is able to reduce the cell viability of HepG-2, MCF-7, and A-549. It is also able to act as an antiproliferative agent against these cell lines, and its activity increases with increasing concentrations. Previously, it has been demonstrated that docosenamide contains essential neuro-signaling molecules and acts as an endogenous bioregulator to regulate and control the formation of tumors, circulatory disorders, inflammation, nociception, anxiousness, and depression (Sonbol et al., 2023). Numerous investigations have shown that docosenamide, sometimes referred to as erucic amide, has anticancer properties (dos Santos et al., 2015). Although research into the precise mechanisms is ongoing, a number of paths have been suggested. It has been demonstrated that docosenamide causes cancer cells to undergo apoptosis, or programmed cell death. Activating signaling pathways that result in cell death is part of this. By stopping the cell cycle at particular checkpoints, docosenamide can stop cancer cells from multiplying and dividing. Docosenamide may prevent the development of new blood vessels, which are necessary for tumor growth and metastasis. It has been demonstrated that docosenamide damages cancer cells’ DNA, resulting in senescence or cell death. It is noteworthy that the precise mechanisms underlying the anticancer activity of docosenamide may differ based on the particular cellular environment and cancer type (Prasathkumar et al., 2022).

Consistent with earlier research findings (Eldehna et al., 2018; Prasathkumar et al., 2022), the current study emphasized that (Z)-13-docosenamide has been demonstrated to have a subsequent biological activity on oxidative stress indicators such as SOD, MDA, CAT, and GSH enzymes that impact HEPG-2 cells, wherein the level of CAT that catabolizes ROS was demonstrated to be reduced in HEPG-2 cells treated with (Z)-13-docosenamide compared to the control, ensuring the subsequent buildup of ROS that ultimately impacts tumor cell death through protein oxidation. These findings support the theory that this drug tries to produce ROS in order to exert its cytotoxic effects. According to these findings, the oxidative damage caused by the excess production of ROS resulted in a decreased quantity of protein in the treated cells compared to the control (Eldehna et al., 2018). Moreover, antioxidant enzymes provide cellular defenses against ROS, which are natural by-products of cellular metabolism. Cells are protected from ROS by these antioxidant enzymes. According to Rahman et al. (2019), the incidence of elevated tumors may cause an increase in these enzymes’ activity. Carcinogenesis is known to be initiated and promoted by lipid peroxidation and free radicals. MDA, a by-product of lipid peroxidation, has been shown to be both carcinogenic and mutagenic. Lipid peroxidation is elevated during the carcinogenic process (Ahmed Amar et al., 2019). H_2_O_2_ is broken down by CAT into H_2_O and OH−. Furthermore, SOD, which is found in many tissues and organs, is also believed to shield cells from superoxide radical damage (Popovici et al., 2021). The electron transfer oxidase system causes biological oxygen reduction, which results in the production of the superoxide radical O_2_. The one-electron reduction of oxygen to the superoxide radical is catalyzed by SOD. SOD reduces the toxicity brought on by the process that prevents free radicals from forming from oxygen (Popovici et al., 2021). In addition, the maintenance of the thiol state of proteins, the elimination of H_2_O_2_, lipid peroxide free radicals, the translocation of amino acids across cell membranes, and the detoxification of ROS are all components of the well-known antioxidant defense mechanism of cells, known as GSH. The present study has proved that (Z)-13-docosenamide has a protective role against cell proliferation by directly reacting with ROS, as demonstrated by the depletion of GSH status in cancer control cells and the increase in (Z)-13-docosenamide treatment. Additionally, GSH activity is increased by (Z)-13-docosenamide, which amply illustrates its protective function against oxidative damage. The data clearly show that (Z)-13-docosenamide increases antioxidant status and inhibits lipid peroxidation. Hence, (Z)-13-docosenamide might work well as a chemotherapy drug from the present results.

In relation to this, molecular docking analysis reveals the distinct mechanisms of action for docosenamide interaction across all three protein–ligand complexes as well as the absence of hydrogen bonding interactions, suggesting that the binding mechanism is predominantly driven by hydrophobic and van der Waals forces. The tubulin beta chain exhibited the strongest binding affinity (−6.1 kcal/mol), followed by OMP-A (−5.5 kcal/mol) and TGF-β II (−3.3 kcal/mol). This pattern indicates that the ligand has varying degrees of specificity for different protein targets, with a particular preference for the tubulin beta chain binding site. The binding site analysis revealed that in all three cases, the ligand interacts with a mix of hydrophobic and polar residues, creating a stable binding environment. The stronger binding affinity observed with the tubulin beta chain can be attributed to the optimal spatial arrangement of hydrophobic residues in its binding pocket, which complements the structure of (Z)-13-docosenamide. The moderate binding energy observed with TGF-β II suggests that while interaction is possible, it may not be the primary target for this compound.

These findings provide valuable insights into the potential biological activity of (Z)-13-docosenamide and its mechanism of action. The varying binding affinities across different protein targets suggest that this compound may exhibit diverse biological effects, with potentially stronger effects on tubulin-dependent processes. The predominance of hydrophobic interactions in all three complexes indicates that the compound’s biological activity is likely mediated through non-polar interactions, which could be important for its therapeutic applications.

## 5 Conclusion

The results of this investigation show the efficiency of (Z)-13-docosenamide to act as an antimicrobial, antioxidant, and anticancer agent. The fact that (Z)-13-docosenamide is a natural substance separated from filamentous fungus *P. chrysogenum* and possesses significant antioxidant activity indicates its safe use for the treatment of AMR microbial infections and hepatocellular carcinomas that were tested. Furthermore, our results could be considered as a positive resource to the pipeline of antimicrobial and anticancer drug discovery. In addition, the outcomes of this study focus on the filamentous fungi as a potential affordable source of bioactive products with several medicinal applications, especially those that would have a role in reducing the global health burden of AMR and the increased cancer-associated deaths.

## Data Availability

The original contributions presented in the study are included in the article/**Supplementary Material**. Further inquiries can be directed to the corresponding authors.

## Supplementary material

Supplementary Table 1 Fungal isolates, preliminary identification, and antioxidant activity of their intracellular and extracellular extracts.

Supplementary Table 2 Antimicrobial activity of crude fungal extracts compared to Gentamycin and Ketoconazole as positive controls.

Supplementary Figure 1 TLC analysis **(A)** TLC purification of the most active fungi (Ft: *Fusarium trivolium*, Af: *Aspergillius flavus*, At: *Aspergillus terreus*, Ao: *Aspergillus ochrachaes*, Pc: *Penicillium chrysogenum*, Cs: *Cladosporium* sp., sp Fo: *Fusarium oxysporium*, An: *Asergillus niveus*, Ps: *Penicillium* sp and Pi: *Penicillium citrinium.*) extracellular extracts **(B)** The TLC purity of the obtained compound sub-fractions from the most active fungus (*P. chrysogenum)* after flash chromatography.

Supplementary Figure 2 GC-Ms analysis for secondary metabolites *P. chyrsogenum* extract.
