# Supplementary material for: Antimicrobial and cytotoxic activities of natural (Z)-13-docosenamide derived from Penicillium chrysogenum
Source: Front Cell Infect Microbiol. 2025 Feb 27;15:1529104. doi: 10.3389/fcimb.2025.1529104 (PMC11903434; doi:10.3389/fcimb.2025.1529104)
Supplement: Supplementary Table 1 — Fungal isolates, preliminary identification, and antioxidant activity of their intracellular and extracellular extracts. [file DataSheet1.docx]

| **Table S1.**Fungal isolates, preliminary identification, and antioxidant activity of their intracellular and extracellular extracts. | | | |
| --- | --- | --- | --- |
| **Isolate code** | **Preliminary iidentification** | **DPPH scavenging %** | |
|  |  | **Intracellular extract** | **Extracellular extract** |
| Control | Ascorbic acid | 92.04±0.49 | 92.04±0.49 |
| Pc | *Penicillium chrysogenium* | 75.87±0.15^a^ | 93.27±0.38 ^a^ |
| Af | *Aspergillus flavus* | 20.30±0.26^b^ | 88.40±0.46 ^b^ |
| At | *Aspergillus terreus.* | 13.17±0.15 ^k^ | 67.97±0.06 ^c^ |
| Ao | *Aspergillius oryza* | 30.33±0.31 ^d^ | 78.27±0.06 ^d^ |
| As | *Alternaria sp.* | - | - |
| Hs | *Helminthosporium sp.* | - | 77.57±0.21 ^e^ |
| Cs | *Cladosporium sp.* | 66.77±0.23 ^f^ | 77.47±0.42 ^f^ |
| Fo | *Fusarium oxysporium.* | 26.37±0.31 ^g^ | 68.80±0.10 ^g^ |
| Au | *Aspergillus fumigatus* | - | 81.50±0.35 ^h^ |
| Rs | *Rhizopus sp.* | - | - |
| Av | *Aspergillus flavapus* | - | 63.60±0.26 ^i^ |
| Us | *Ulocladium sp.* | - | 47.40±0.10 ^j^ |
| An | *Aspergillus niveus* | 13.17±0.15 ^k^ | 56.60±0.26 ^k^ |
| Ns | *Cunninghamella sp.* | - | - |
| Th | *Trichoderma harezanium* | - | 60.40±0.10 ^l^ |
| Ac | *Aspergillus ochracous.* | 13.57±0.15 ^m^ | - |
| Bs | *Beauvaria sp.* | - | 32.37±0.25 ^n^ |
| Ab | *Aspergillus carbonarius.* | - | 68.70±0.26 ^o^ |
| Ms | *Mucor sp.* | - | 41.40±0.10 ^p^ |
| Ps | *Penicilium sp.* | 33.37±0.15 ^q^ | 56.47±0.21 ^q^ |
| Ts | *Trichoderma sp.* | - | 67.43±0.42 ^r^ |
| Pi | *Penicillium citrinium.* | 45.53±0.21 ^s^ | 25.43±0.15 ^s^ |
| Fm | *Fusarium moniliforium.* | - | - |
| Ag | *Aspergillus niger.* | - | - |
| Ft | *Fusarium trivolium.* | 11.47±0.21 ^t^ | 18.60±0.20 ^t^ |
| Data are the calculated mean of 3 replicates ± standard deviation (SD). Different letters in the same column denote significant differences at the p < 0.05 level when compared pairwise of different fungal strain with control using an Independent t-test. | | | |

| **Table S2.** Antimicrobial activity of crude fungal extracts compared to Gentamycin and Ketoconazole as positive controls. | | | | | | | | | | | | | |
| --- | --- | --- | --- | --- | --- | --- | --- | --- | --- | --- | --- | --- | --- |
| **Isolate code** | | Bacterial strains | | | | | | | | Fungal strains | | | |
|  |  | ***S. aureus*** | | ***B. subtilis*** | | ***E. coli*** | | ***K. pneumoniae*** | | ***A. fumigatus*** | | ***P. aurantiogriseum*** | |
|  |  | **Intra** | **Extra** | **Intra** | **Extra** | **Intra** | **Extra** | **Intra** | **Extra** | **Intra** | **Extra** | **Intra** | **Extra** |
| **Fungal isolates** | Pc | 12.17±0.15^a,b*^ | 39.23±0.25 ^a,b*^ | 10.50±0.44 ^a,b*^ | 37.33±0.35 ^a,b*^ | 9.10±0.10 ^a,b^ | 38.43±0.45 ^a,b*^ | - | 35.10±0.10 ^a,b*^ | - | 33.37±0.15 ^a,b^ | 6.13±0.15 ^a ,b^ | 36.50±0.40 ^a,b*^ |
|  | Af | - | 37.17±0.21 ^a,b,c^ | 6.10±0.10 ^a,b,c^ | 33.17±0.29 ^b,c^ | - | 32.17±0.21 ^a,b ,c^ | 10.13±0.23 ^a,b*^ | 30.13±0.15 ^a,b,c^ | - | 31.47±0.35 ^a,b ,c^ | - | 33.43±0.40 ^a,b,c^ |
|  | At | 10.20±0.20^a,b,c^ | 30.10±0.17 ^a,b,c ,d^ | - | 31.02±0.02 ^a,b,c ,d^ | 8.17±0.15 ^a,b,c^ | 29.40±0.40 ^a,b,c,d^ | - | 28.10±0.17 ^a,b,c,d^ | 9.20±0.20 ^a,b^ | 29.50±0.17 ^a,b,c,d*^ | 11.23±0.20 ^a,b,c*^ | 30.37±0.38 ^a,b,c ,d^ |
|  | Ao | - | 26.13±0.15 ^a,b,c,d,e^ | 6.17±0.15 ^a,b,d^ | 28.17±0.29 ^a,b,c,d ,e^ | 15.20±0.20 ^a,b,c ,d*^ | 24.30±0.30 ^a,b,c,d,e^ | 9.13±0.12 ^a,b,c^ | 22.97±0.06 ^a,b,c,d,e^ | 12.27±0.25 ^a,b,c^ | 22.50±0.10 ^a,b,c ,d,e^ | - | 29.27± 0.46 ^a,b,c,e^ |
|  | As | 3.20±0.26 ^a,b,c,d^ | 29.20±0.17 ^a,b,c,d,e,f^ | - | 22.17±0.15 ^a,b,c ,d,e,f^ | - | 23.13±0.23 ^a,b,c,d,e,f^ | - | 20.53±0.23 ^a,b,c,d,e ,f^ | - | 23.30±0.26 ^a,b,c,d,f^ | - | 19.03±0.06 ^b,c,d,e,f^ |
|  | Hs | 1.87±0.32 ^a,b,c,d,e^ | 18.17±0.29 ^a,b,c ,d,e,f,g^ | 5.17±0.29 ^a,b,c,e^ | - | - | 20.13±0.23 ^a,b,c,d,e,f,g^ | - | 22.17±0.15 ^a,b,c,d,e,f,g^ | - | 17.17±0.15 ^a,b,c ,d,e,f,g^ | - | 25.37±0.32 ^a,b,c ,d,e,f,g^ |
|  | Cs | 2.13±0.23 ^a,b,c,f^ | 22.07±0.12 ^a,b,c ,d,e,f,g,h^ | - | 15.23±0.25 ^a,b,c ,d,e,f,g^ | - | 24.10±0.17 ^a,b,c,d,f,g,h^ | - | 20.03±0.06 ^a,b,c,d,e,g,h^ | 5.10±0.10 ^a,b,c,d^ | 18.10±0.10 ^b,c ,d,e,f,g,h^ | 4.07±0.11 ^a,b,c,d,e^ | - |
|  | Fo | - | 17.13±0.15 ^a,b,c ,d,e,f,g,h,i^ | - | 20.03±0.06 ^a,b,c ,d,e,f,g,h^ | 4.47±0.11 ^a,b,c,d,e^ | - | 7.33±0.11^a,b,c,d^ | 16.17±0.15 ^a,b,c,d,e,f,g,h,i^ | - | - | - | 23.30±0.26 ^a,b,c ,d,e,f ,g,h^ |
|  | Au | - | 36.10±0.10 ^a,b,c ,d,e,f,g,h,I,j^ | - | 23.10±0.10 ^a,b,c ,d,e,f,g,h,i^ | - | 24.04±0.06 ^a,b,c,d,f,g,i^ | - | 25.10±0.10 ^b,c ,d,e,f,g,h,I,j^ | - | 25.13±0.23 ^a,b,c ,d,e,f,g,h,i^ | - | 19.33±0.29 ^b,c,d,e,g,h,i^ |
|  | Rs | 7.23±0.25 ^a,b,c,d,e,g^ | 28.10±0.17 ^a,b,d,e,f,g,h,I,j,k^ | - | - | - | 20.73±0.15 ^a,b,c,d,e,f,h,I,j^ | - | 18.17±0.29 ^a,b,c ,d,e,f ,g,h,I,j,k^ | 6.53±0.46 ^a,b,c,d,e^ | - | 3.13±0.23 ^a,b,c,d^ | - |
|  | Av | - | 30.13±0.15 ^a,b,c ,d,e,f,g,h,I,j,k,l^ | 7.13±0.15 ^a,b,c,d,f^ | 19.37±0.32 ^a,b,c ,d,e,f,g,I,j^ | 11.43±0.06 ^a,b,c,d,e^ | - | - | - | 12.30±0.30 ^a,b,d,e,f^ | 20.30±0.26 ^a,b,c ,d,e,f,g,h,I,j^ | - | 28.27±0.46 ^a,b,c,d, f,g,h,I,j^ |
|  | Us | - | 19.93±0.06 ^a,b,c ,d,e,f,g,h,I,j,k,l,m^ | - | 16.23±0.21 ^a,b,c ,d,e,f,g,h,I,j,k^ | - | 20.13±0.15^a,b,c,d,e,f,h,I,k^ | - | 16.17±0.15 ^a,b,c ,d,e,f ,g,h,j,k,l^ | - | 17.27±0.46 ^a,b,c ,d,e,f,I,j,k^ | - | 22.83±0.11 ^a,b,c,d,e,f,g,I,j,k^ |
|  | An | - | 20.10±0.10 ^a,b,c ,d,e,f,g,h,I,j,k,l,n^ | - | 15.20±0.17 ^a,b,c ,d,e,f,g,h,i,j,l^ | - | 18.17±0.15 ^a,b,c,d,e,f,g,h,i,j,k,l^ | - | 17.17±0.28 ^a,b,c ,d,e,f,g,h,i,j,k,l,m^ | - | - | - | - |
|  | Ns | - | 16.17±0.21 ^a,b,c ,d,e,f,g,h,I,j,k,l,m,n,o^ | 7.67±0.40 ^a,b,c ,d,e,g^ | - | - | - | - | - | - | 16.23±0.21 ^a,b,c ,d,e,f,g,h,i,j,k,l^ | - | 13.50±1.32 ^a,b,c,d,e,f,g,h,I,j,k,l^ |
|  | Th | - | - | - | 18.20±0.17 ^a,b,c,d,e,f,g,h,i,j,k,m^ | - | 18.10±0.10 ^a,b,c,d,e,f,g,h,i,j,k,m^ | - | 20.13±0.15 ^a,b,c,d,e,g,i,j,k,l,m,n^ | - | - | - | - |
|  | Ac | - | - | - | 14.43±0.39 ^a,b,c ,d,e,f,g,h,i,j,k,l,n^ | - | 14.23±0.25 ^a,b,c,d,e,f,g,h,i,j,k,l,m,n^ | - | - | - | - | - | - |
|  | Bs | 9.30±0.26^a,b,c,d,e,f,h^ | - | 3.13±0.23 ^a,b,c ,d,e,f,h^ | 13.20±0.35 ^a,b,c ,d,e,f,g,h,i,j,k,l,m,o^ | - | - | 5.47±0.42 ^a,b,c,d^ | - | - | 21.27±0.25 ^a,b,c ,d,e,f,g,i,j,k,l,m^ | - | 18.27±0.23 ^b,c,d,e,g,h,j,k,m^ |
|  | Ab | - | 27.97±0.06 ^a,b,c ,d,e,f,g,h,I,j,l,m,n,o,p^ | - | 18.30±0.26 ^a,b,c ,d,e,f,g,h,i,j,k,m,n,p^ | - | 21.23±0.21 ^a,b,c,d,e,f,g,h,i,k,l,m,n,o^ | - | 24.27±0.25 ^a,b,c ,d,e,f,g,h,i,j,k,l,m,n,o^ | 12.40±0.35 ^a,b,d,e^ | - | - | - |
|  | Ms | - | 20.10±0.10 ^a,b,c ,d,e,f,g,h,I,j,k,l,o,p,q^ | - | - | - | 14.13±0.15 ^a,b,c,d,e,f,g,h,i,j,k,l,m,p^ | - | 18.14±0.14 ^a,b,c ,d,e,f ,g,h,i,j,l,m,n,o,p^ | - | 17.43±0.45 ^a,b,c ,d,e,f,i,j,l,m,n^ | - | - |
|  | Ps | - | 25.10±0.17 ^a,b,c ,d,e,f,g,h,I,j,k,l,m,n,o,p,q,r^ | - | 20.17±0.21 ^a,b,c ,d,e,f,g,i,j,k,l,m,n,o,q^ | - | 23.10±0.10 ^a,b,c,d,e,g,h,j,k,l,m,n,q^ | - | 22.17±0.21 ^a,b,c ,d,e,f,h,i,j,k,l,m,n,o,p,q^ | - | - | - | - |
|  | Ts | - | 13.23±0.21 ^a,b,c ,d,e,f,h,I,j,k,l,m,n,p,q,r,s^ | - | 12.40±0.10 ^a,b,c ,d,e,f,g,h,i,j,k,l,m,n,o,p,r^ | - | 15.17±0.15 ^a,b,c,d,e,f,g,h,i,j,k,l,m,n,o,r^ | - | 18.10±0.10 ^a,b,c ,d,e,f ,g,h,i,j,m,n,o,r^ | - | - | - | - |
|  | Pi | - | 14.27±0.25 ^a,b,c ,d,e,f,g,h,I,j,k,l,m,n,o,p,q,r,s,t^ | - | - | - | - | - | 16.40±0.36 ^a,b,c,d,e,f,g,h,i,j,k,l,m,n,o,p,r,s^ | - | - | - | - |
|  | Fm | - | - | - | - | - | 12.23±0.21 ^a,b,c,d,e,f ,h,i,j,k,l,m,n,o,s^ | - | - | - | 13.13±0.23 ^a,b,c ,d,e,f,g,h,i,j,k l,m,n^ | - | - |
|  | Ag | - | 17.10±0.10 ^a,b,c ,d,e,f,g,h,I,j,k,l,m,n,o,p,q,r,s,t^ | - | - | - | - | - | 11.37±0.32 ^a,b,c,d,e,f,g,h,i,j,k,l,m,n,o,p,q,r,s^ | - | - | - | 15.13±0.2 ^a,b,c,d,e,f,g,h,i,j,k,m,n^ |
|  | Ft | 9.10±0.10 ^a,b,c ,d,e,f^ | - | 5.23±0.21 ^a,b,d,e,f,g^ | 13.07±0.12 ^a,b,c ,d,e,f,g,h,i,j,k,l,m,o,p^ | - | 12.17±0.15^a,b,c,d,e,f,h,i,j,k,l,m,n,o^ | - | - | - | - | - | 10.30±0.26 ^a,b,c,d,e,f,g,h,i,k,m,n^ |
| **Positive controls** | Gen. | 11.10±0.17 **^a^** | | 33.23±0.21 **^a^** | | 19.47±0.15 **^a^** | | 25.43±0.38 **^a^** | | - | | - | |
|  | Keto. | - | | - | | - | | - | | 18.43±0.45 **^a^** | | 19.23±0.21 **^a^** | |
| Intra: refers to intracellular extract.  Extra: refers to extracellular extract.  Gen.: Refers to Gentamycin.  Keto.: refers to Ketoconazole.  Data are the mean of 3 replicates ± standard deviation (SD). a: indicate significant differences at the p < 0.05 level when comparing all fungal strains with control 1 or 2.  b-s: indicate significant differences at the p < 0.05 level when pairwise comparison of fungal strains.  *: Indicate highly significant differences among the fungal strains and control using multiple comparisons according to one-way ANOVA test. | | | | | | | | | | | | | |


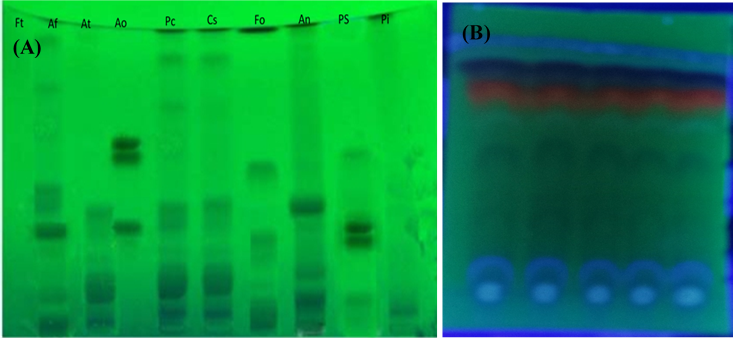


**Figure S1.**TLC analysis **(A):** TLC purification of the most active fungi (Ft: *Fusarium trivolium*, Af: *Aspergillius flavus*, At : *Aspergillus terreus*, Ao: *Aspergillus ochrachaes* ,

Pc: *Penicillium* *chrysogenum* , Cs: *Cladosporium sp,* Fo: *Fusarium oxysporium*, An: *Asergillus niveus*, Ps: *Penicillium sp* and Pi: *Penicillium citrinium.*) extracellular extracts

**(B):** The TLC purity of the obtained compound sub-fractions from the most active fungus (*P. chrysogenum)* after flash chromatography.


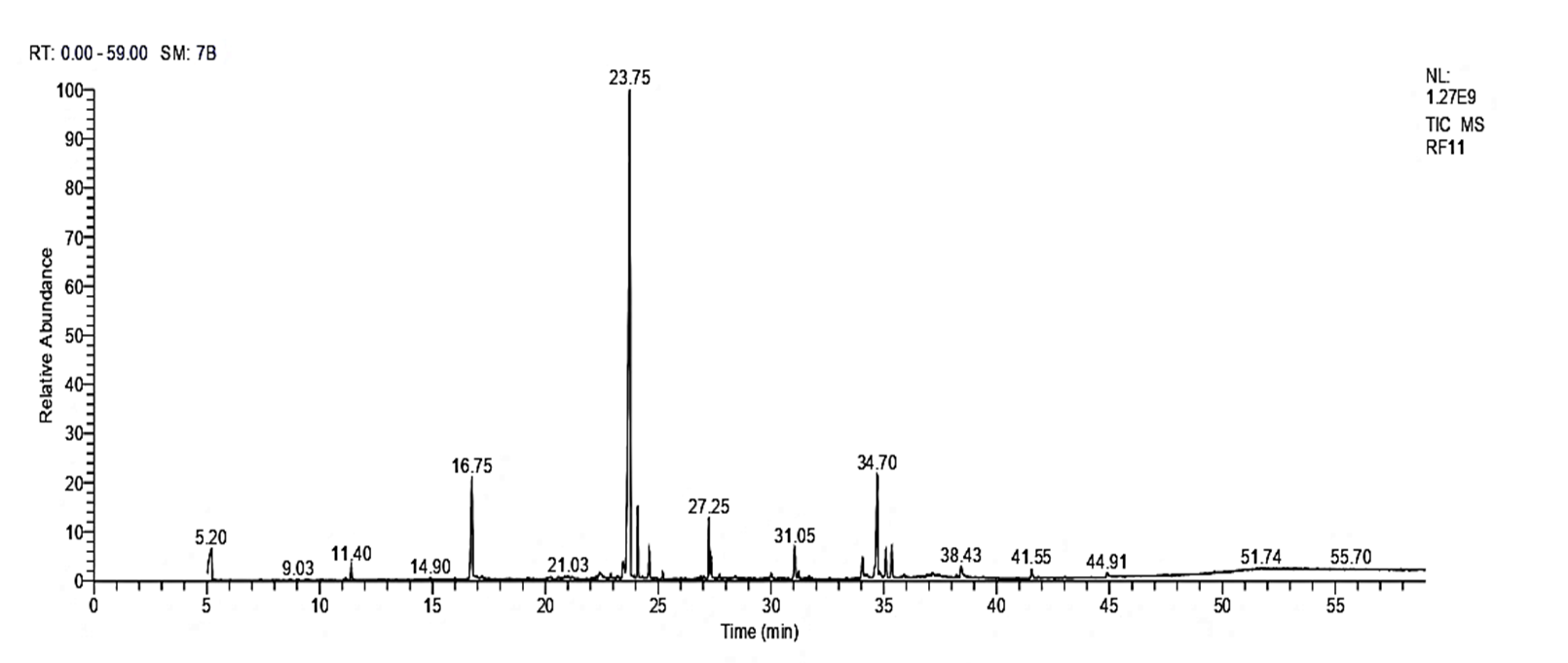


**Figure S2.** GC-Ms analysis for secondary metabolites *P. chyrsogenum* extract
